# Induction of PD-1 and CD44 in CD4^+^ T cells by circulatory extracellular vesicles from severe dengue patients drives endothelial damage via the NF-kB signaling pathway

**DOI:** 10.1128/jvi.01861-24

**Published:** 2024-12-31

**Authors:** Sharda Kumari, Ankit Biswas, Tushar Kanti Maiti, Bhaswati Bandyopadhyay, Arup Banerjee

**Affiliations:** 1 Laboratory of Virology, Regional Centre for Biotechnology, National Capital Region Biotechnology Science Cluster 682813 , Faridabad, Haryana, India; 2 Functional Proteomics Laboratory, Regional Centre for Biotechnology, National Capital Region Biotechnology Science Cluster 682813 , Faridabad, Haryana, India; 3 Department of Microbiology, School of Tropical Medicine 76063 , Kolkata, West Bengal, India; University of Kentucky College of Medicine, Lexington, Kentucky, USA

**Keywords:** extracellular vesicles, CD44, PD-L1 dengue, endothelial cells

## Abstract

Extracellular vesicles (EVs) emerged as critical contributors to the pathogenesis of vascular endothelial barrier dysfunction during the inflammatory response to infection. However, the contribution of circulating EVs to modifying endothelial function during dengue virus infection remains unclear. In this study, we showed that severe dengue patients’ plasma-derived EV (SD-EV) were found to carry elevated levels of different protein cargos, e.g., immunoregulatory proteins (PD-L1, CD44). Further, we demonstrated that SD-EV induces PD-1 and CD44 expression on CD4^+^ T cells. SD-EV-modulated CD4^+^ T (SD-EV-CD4) cells released secretome delayed endothelial cell (EC) migration, arrested them in the G1 phase, and augmented the expression of PD-L1 and ICAM-1 expression on EC through the Notch signaling pathway. Blocking SD-EV and CD4^+^ T-cell interaction through the PD-1/PD-L1 pathway partially rescued the CD4^+^ T cell’s effect on EC but did not alter ICAM-1 expression on EC. We observed that the ICAM-1 expression on EC and hyaluronic acid (HA) release from EC was mediated by CD44, which was elevated on SD-EV-modulated CD4^+^ T cells (SD-EV-CD4), indicating a permeability defect. Blocking of CD44 on SD-EV-CD4 significantly reduced ICAM-1 expression on EC. Further, depletion of specific cytokines, e.g., TNF-α and not IFN-γ from the SD-EV-CD4 secretome, reduced ICAM-1 expression, decreased transendothelial electrical resistance, and induced apoptosis on EC significantly. Treatment with NF-kB inhibitor before secretome addition to EC reduced ICAM-1 expression on EC. In conclusion, we provided evidence that SD-EV-CD4 carrying PD-1 and CD44, when interacting with EC, significantly affected endothelial cell properties and may be significant in dengue-mediated endothelial dysfunction.

**IMPORTANCE:**

Extracellular vesicles (EVs) are small membrane vesicles secreted into biological fluids, including plasma from living cells, holding insights into pathological processes. Studying EVs under pathological conditions is extremely important as they play a selective role in intercellular communication and modulation of immune response under diverse pathological conditions. However, there is less clarity on how circulatory extracellular vesicles influence immune cells during dengue virus (DV) infection and impact pathogenesis. Our present study highlights the impact of severe dengue patients’ plasma-derived EV (SD-EV) on CD4^+^ T cells and together induce endothelial barrier dysfunction. We provided evidence that SD-EV induces PD-1 and CD44 on CD4^+^ T cells and, when interacting with endothelial cells (EC), drives endothelial damage through direct interaction or secretome and may be significant in dengue-mediated endothelial dysfunction.

## INTRODUCTION

Dengue virus (DV) belongs to the Flaviviridae family, spreads through mosquito bites, and is mainly associated with a self‐limiting illness. However, it can cause severe clinical disease manifestations, such as dengue hemorrhagic fever (DHF) and severe dengue (SD). Recent reports by WHO suggest that over 7.6 million dengue cases have been reported in 2024, including 3.4 million confirmed cases, over 16,000 severe cases, and over 3,000 deaths (https://www.who.int/emergencies/disease-outbreak-news/item/2024-DON518). Reduced platelet counts and endothelial dysfunction leading to vascular leak are the hallmarks of severe dengue (1). An increase in vascular permeability typically becomes clinically evident 3–6 days after the onset of illness. This results in the accumulation of fluid in pleural and peritoneal cavities and a reduction in blood pressure and pulse pressure, resulting in poor organ perfusion. Finally, patients die due to hypovolemic shock (2). Although endothelial cells represent the primary fluid barrier of the blood vessels, the extent to which these cells contribute to DV pathology is still under debate. Several evidences suggest that vascular leakage is likely to occur due to inflammatory mediators rather than direct infection of the endothelium (3, 4). Reports are also available on the involvement of dengue-soluble protein, e.g., NS1, in disrupting the endothelial glycocalyx and thus contributing to vascular leak (5, 6). However, there is a disagreement between the timings of the peak in viral load and NS1 antigenemia and vascular leakage (7). Also, patients with secondary infection are at higher risk for developing SD through antibody-dependent enhancement and promoting viral replication, which, in turn, accelerates vascular permeability and other complications (8).

Cytokines, such as tumor necrosis factor‐*α* and many other inflammatory lipid mediators*,* e.g., platelet-activating factor (PAF), known to be elevated in the critical phase of dengue, are likely contributing factors (9, 10). Platelets have also been shown to significantly contribute to endothelial dysfunction by inducing the production of inflammatory cytokines by monocytes through activation of NLRP3 inflammasome (11, 12). Besides the proinflammatory mediators, platelet (Plt)-derived extracellular vesicles (Plt-EVs) can regulate endothelial cell permeability (13). However, the role of circulating EVs in endothelial barrier dysfunction in dengue infection has not yet been reported.

The interaction between T cells and endothelial cells (ECs) is also important. It is necessary for the inflammatory response, allowing T cells to extravasate from the circulation and migrate to infectious or sterile inflammation sites (14). EC express class I and class II major histocompatibility complex (MHC)–peptide complexes on their surface along with costimulatory and co-inhibitory molecules, such as intercellular adhesion molecule-1 (ICAM-1), vascular cell adhesion molecule-1 (VCAM-1), ICOS-L (CD275), and PD-L1 (15). On the other hand, T cells express ligands/receptors of endothelial adhesion molecules to sustain these interactions that eventually result in T-cell extravasation into sites of inflammation. Haltaufderhyde et al. reported a significant PD-1 expression on CD8 and CD4 T cells during the febrile, critical, and early convalescence phases of dengue infection (16). Cytokines produced by activated T cells can trigger endothelial damage (17). Therefore, the endothelial cell behavior may change depending on the ligand expressed on the T cells.

Vascular endothelial cells cover the innermost wall of arteries, capillaries, and veins, which is decisive in preserving the barrier function. However, inflammatory stimuli can breach the endothelial barrier, which is strongly linked to plasma leakage observed in dengue infection. The PD-1/PD-L1 axis has been greatly investigated in various organ-associated endothelial cells (18, 19); however, limited studies are available describing the contribution of this pathway to vascular endothelium damage.

Our group recently reported an interaction of circulating EVs derived from the plasma of SD patients with T cells, resulting in CD4^+^ T-cell suppression through PD-L1/PD-1 interaction (20). Interestingly, SD is associated with increased release of platelet extracellular vesicles in plasma. However, the outcome of EV-CD4 interaction on EC properties was not clear. Therefore, we conducted a study to understand the effect of dengue plasma-derived EV and EV-modulated CD4^+^ Tcell interaction on ECs. We found EV alone, EV-modulated CD4^+^ T cells, and its supernatant via TNF-α impacted different properties of endothelial cells.

## RESULTS

### Characterization of plasma-derived extracellular vesicles from dengue patients

In this study, we used plasma samples from healthy donors (HDs), dengue-negative other febrile illness (OFI), mild dengue (MD), and severe dengue (SD) to investigate the effect of plasma-derived EV on endothelial cellular proliferation, migration, and associated mechanisms.

**Fig 1:**
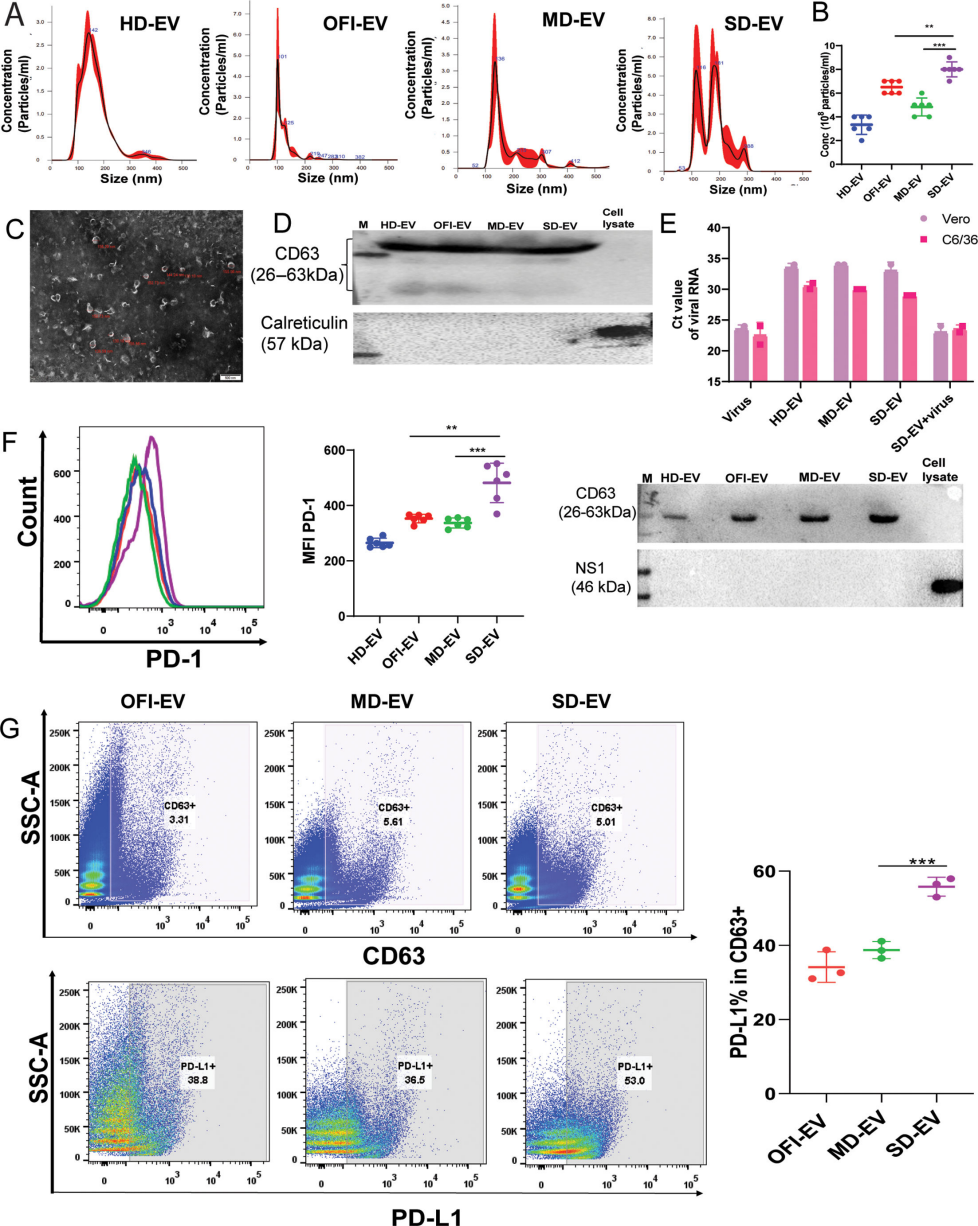
Characterization of EV isolated from plasma. (**A**) Nanoparticle tracking analysis (NTA) graph representing concentration (particles/mL) and particle size distribution of plasma-derived extracellular vesicles (EVs). Each particle’s Brownian motion was tracked thrice between frames. (**B**) The concentration (particles/mL) of EVs of plasma is shown. Each dot represents one set of EVs. Tukey multiple comparison tests were used. (**P = 0.0066,***P < 0.0001). (**C**) Morphological analysis of plasma EVs was performed using transmission electron micrograph (TEM). EVs appeared cup-shaped at 80 Kv threshold and scale of 500 nm. (**D**) Western blot of EVs was performed after loading 20 µg protein, and CD63 and calreticulin were plotted. (**E**) Cells were incubated with either EV or EV with virus or virus alone, as mentioned in the Materials and Methods section. Viral RNA detection through RT-PCR and cycle threshold (Ct) was plotted. A lower Ct value means a high viral load. Ct value of more than 30 is considered negative for viral RNA. The lower panel represents Western blots for viral NS1 protein and CD63. (**F**) Representative image of MFI of PD-1 on CD4^+^ T cells after incubation with EV for 6 days (left panel). After incubating with EVs from different groups, the combined data graph of PD-1 expression on CD4^+^ T cells (right panel). The Student’s unpaired *t*-test is used to observe the *P*-value (***P = 0.0007; **P = 005). Each dot represents one set of EVs on CD4^+^ T cells. (**G**) A representative image of CD63-PD-L1 is on the surface of the EVs through FACS. The combined data graph of CD63-PD-L1 % of all different groups of EVs (right panel). Each dot represents one set of EVs. The Student’s unpaired *t*-test was used (***P = 0.0003). HD-EV = EV derived from healthy donors, OFI-EV = EV derived from other febrile illness patients, MD-EV = EV derived from mild dengue patients, and SD-EV = EV derived from severe dengue patients.

EVs from plasma were isolated by ultracentrifugation methods and characterized via nanoparticle tracking analysis (NTA), transmission electron micrograph (TEM), and Western blot. NTA and TEM studies confirmed the size and concentration of vesicles purified from plasma samples. All the plasma-derived EVs ranged between 60 and 200 nm, and depending upon the source of EV, concentration ranges from (4 × 10^8^ particles to 7 × 10^8^ /mL of plasma) (Fig. 1A through C). CD63 was used as a marker for EV. In contrast, calreticulin, a chaperone protein found in the endoplasmic reticulum, was used to assess the purity of the EV sample. In Fig. 1D, the expression of CD63 protein was shown, and all EVs isolated from different groups were shown positive signals for CD63. Calreticulin is only found in the cell lysate and not the purified EV fraction, suggesting that the EV fraction is free of contaminating proteins originating through Golgi and ER trafficking.

Several reports suggest co-purifying the virus with EVs (21, 22). Since the EVs were isolated from dengue-infected patients' plasma, we checked if EV purified through ultracentrifugation methods also contained infectious viral particles or viral RNA. We incubated C6/36 with EV isolated from HD, MD, SD, or SD-EV + virus for 2 h. The dengue virus was added to C6/36 cells as a positive control. After 2 h incubation, cells were washed several times, and fresh media were added. Cells were harvested 4 days post-infection (dpi), and intracellular viral RNA from C6/36 cells was checked. Also, cell supernatant collected from EV-treated C6/36 cells was added to Vero cells, and cellular RNA was extracted after 3 dpi. The qRT-PCR and Western blots were performed to detect viral RNA and proteins. In both cases, viral RNA was not detectable in cells incubated with MD or SD-EV (Fig. 1E, upper panel). However, viral RNA and protein were detectable in cells incubating with only virus or SD-EV + virus, suggesting that plasma-derived EVs were devoid of infectious viral particles or RNAs (Fig. 1E, upper and lower panels). Also, we checked the presence of NS1 in EVs derived from mild and severe dengue samples, but we did not find the expression of NS1 in them (Fig. 1E, lower panel). Dengue NS1 protein was detectable in DV-infected C6/36 cell lysate. Thus, our data suggest that the EVs derived from mild and severe dengue patients’ plasma were devoid of viral RNA and its protein.

SD-EV incubated with CD4^+^ T cells for 6 days enhanced the receptor expression PD-1 on their surface (Fig. 1F). Also, through FACS, we confirmed that SD-EV expressed a significantly high level of PD-L1 marker on the surface of EVs (Fig. 1G). These data further reconfirmed our previous observation (20) that SD-EV induces PD-1 expression on CD4^+^ T cells.

### Dysregulation of plasma-derived EV proteome due to dengue infection

**Fig 2:**
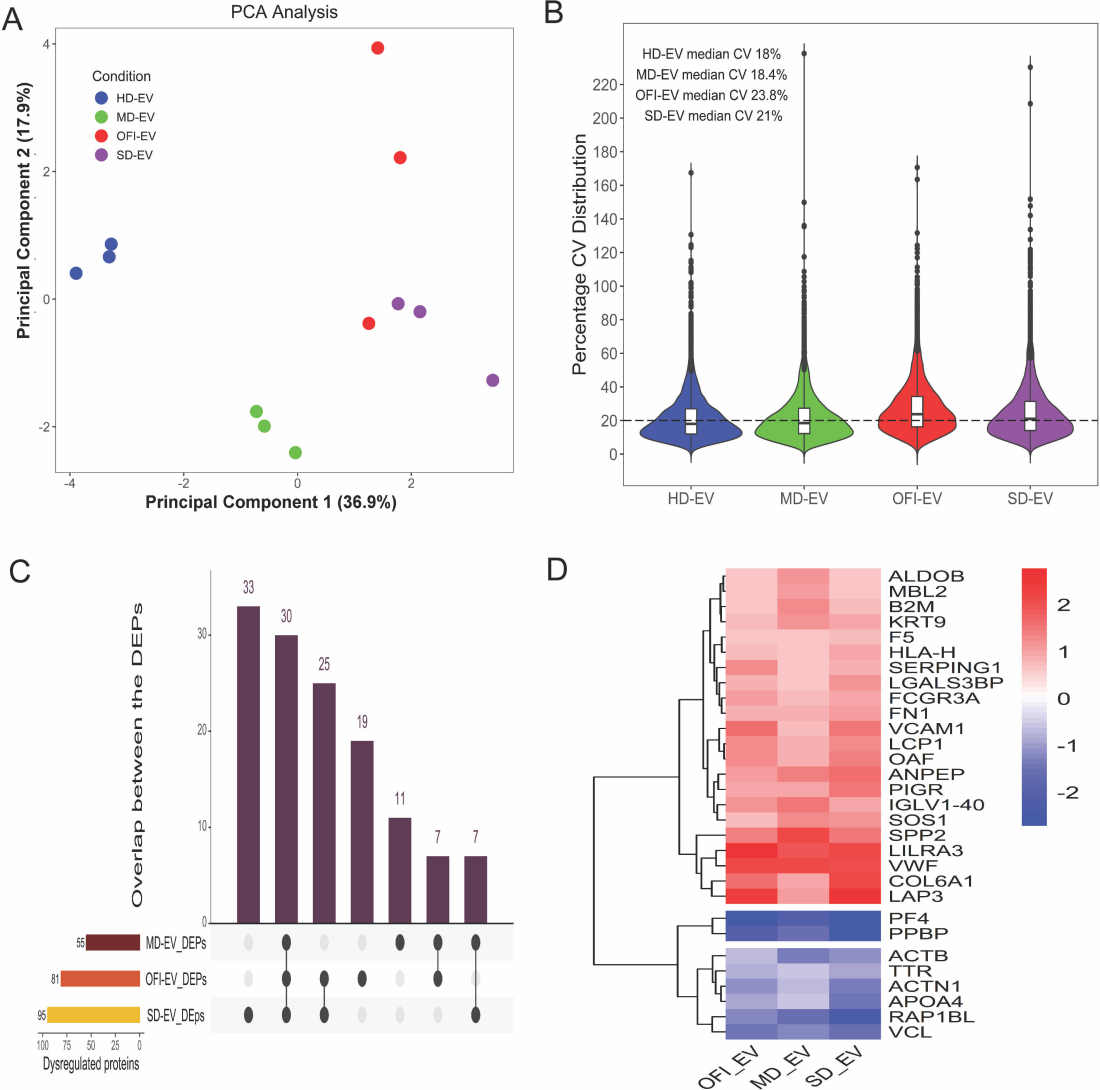
Characterization of dengue patients’ plasma-derived extracellular vesicle proteome. Plasma-derived EVs were identified using the ultracentrifugation method. Proteins were extracted and subjected to mass spectrometry analysis. (**A**) Principal component analysis of samples clustered based on their comparison group separated by PC1 at the x-axis and PC2 at the y-axis, (**B**) Percentage CV distribution of precursors identified in samples from different comparison groups, and (**C**) differential protein expression between the groups is plotted. (**D**) Heatmap of 30 differentially expressed proteins across dengue negative (OFI-EV), mild (MD-EV), and severe (SD-EV) groups.

To examine the proteomic profile of EVs isolated from plasma of different groups, liquid chromatography with tandem mass spectrometry (LC-MS/MS) analysis was performed (Fig. 2). An experiment was set up for comparative proteomic analysis of circulating EV isolated from plasma of HD, OFI, and dengue-infected patients, and differentially expressed proteins were identified.

The proteome analysis of extracellular vesicles identified 284 protein groups across the samples. The expression pattern of these identified proteins across samples can distinctly separate healthy groups from mild samples based on principle component analysis. There are certain overlaps between severe and dengue negative (DN) OFI samples when clustered using PC1 and PC2 with the calculated variation of 36.9% and 17.9%, respectively (Fig. 2A). The Pearson correlation coefficient also suggested a similar observation. The samples from the healthy and mild groups clustered distinctively with a very high correlation coefficient, but there was no separation between samples from OFI and severe dengue groups (Fig. 2A).

We further checked the distribution of protein expression for each sample. We made the violin plot of the proteomics data to capture the variations between sets within each group. The average median value for precursor CV across the comparison group is around 20%, suggesting the separation of groups as actual biological signatures based on their altered proteome (Fig. 2B).

After confirming good data quality (Fig. 2B), we analyzed differential protein expression between the comparison groups. When compared with healthy samples, the mild group yielded 43 upregulated and 12 downregulated proteins (Fig. 2C). Similarly, when comparing severe phenotype with healthy individuals, we found that 18 proteins were downregulated, and 77 proteins were upregulated (Fig. 2C). Finally, we discovered 55 elevated and 26 downregulated proteins in the dengue negative (OFI) vs healthy comparison group (Fig. 2D). Further, the differentially regulated proteins were studied in a group to reveal 30 differentially expressed proteins common across all the comparison groups (Fig. 2C). Among these 30 proteins, 22 were elevated. At the same time, only eight were down (Fig. 2D).

**Fig 3:**
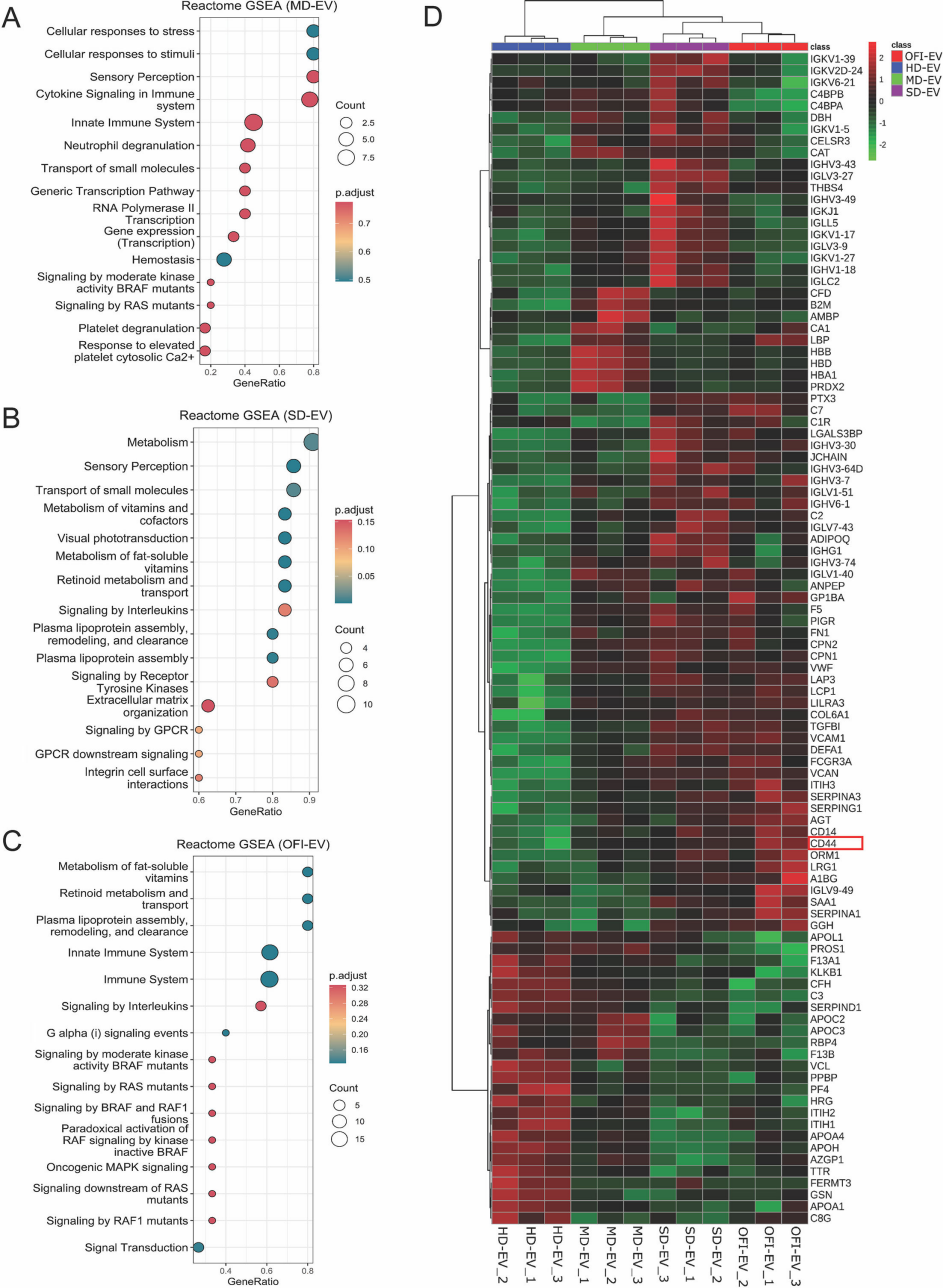
Dysregulated proteins associated with enriched pathway dengue. (**A–C**) Pathway enrichment analysis of dysregulated proteins found in MD-EV, SD-EV, and OFI-EV. (**D**) Heatmap of different proteins found in various groups. The log2 transformed peptide peak intensity was plotted. (Three sets of EV from all groups were used.)

Next, we performed gene set enrichment analysis to identify dysregulated pathways that affect differentially expressed proteins. In mild conditions, cellular response to stress and stimuli, cytokine signaling, innate immune system, and neutrophil degranulation is altered (Fig. 3A). When regulated pathways associated with severe phenotype are investigated, we found that metabolism, metabolism of vitamins, fat-soluble vitamin and cofactor metabolism, retinoid metabolism and transport, and plasma lipoprotein assembly are significantly modulated (Fig. 3B). The differential proteins from OFI groups enriched pathways related to the immune system and signaling by interleukins uniquely (Fig. 3C).

Next, we visualized the expression pattern of the top 100 differentially altered proteins across the samples to identify distinct regulation clusters between the comparison groups (Fig. 3D). After careful inspection, we found apparent alteration of CD14 and CD44 in severe (SD-EV) and OFI but not in the samples with a mild phenotype.

### Severe dengue plasma-derived EV (SD-EV) retarded proliferation and impaired the migratory ability of endothelial cell

**Fig 4:**
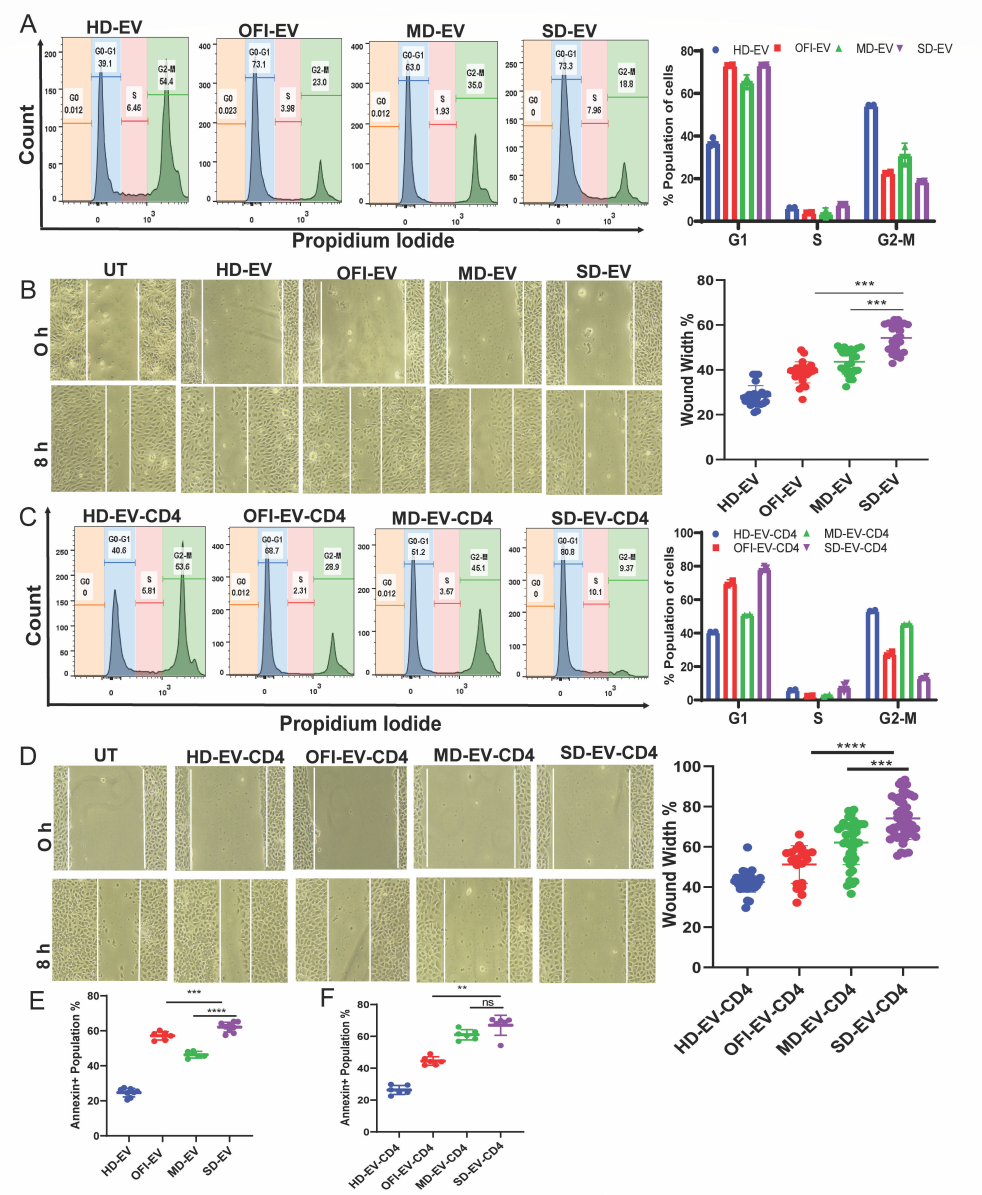
Functional role of EV and EV-modified CD4^+^ T cells on endothelial cells. (**A**) Representative cell cycle analysis of endothelial cells (EC) after incubation with EV for 18 h using propidium iodide. The right panel represents combined data of different phases of cell cycle analysis of all sets of EV with EC. (**B**) A cell migration assay of ECs was performed by giving a wound to these cells by 200 µL tip and taking images at 0 h and after 8 h of incubation (left panel). These wounded endothelial cells were incubated with EV. The combined graph is plotted for the wound width percentage, where initial wound width was taken as 100%, and after incubation measurements, percentages were plotted (right panel). Student’s unpaired *t*-test is used. (**C**) Representative analysis of cell cycle analysis of EC after incubation with EV-modulated CD4^+^ T cells for 18 h using propidium iodide (left panel). The right panel represents combined data of cell cycle analysis of all groups of EV-modulated CD4^+^ T cells with EC. (**D**) Representative images of EC cell migration assay after 8 h incubation of EC with EV-modulated CD4^+^ T cells (left panel). The quantitative graphs are plotted percentage-wise, which showed the least cell migration ability/enhanced wound width % in EC in the presence of SD-EV-modulated CD4^+^ T cells. The right panel shows the combined graph of wound width percentage of all four groups. Student’s unpaired *t*-test is used. (**E**) The combined graphs represent data (a percentage of the Annexin V + population) from nine sets of EV from all four groups showing increased apoptosis in the presence of SD-EV compared with other EV. (**F**) The graph represents data from three individual donors showing increased apoptosis in the presence of SD-EV-modified CD4^+^ T cells compared with other EV-modulated CD4^+^ T cells. Student’s unpaired *t*-test is used to calculate the significance. (***P < 0.001) (****P < 0.0001).

Cell cycle analysis was performed to investigate the effect of EVs on endothelial cells. The DNA content was determined by flow cytometry, and the cell subpopulations (G0/G1, S, and G2/M) were calculated. In the HD-EV group, the G0/G1 subpopulation percentage decreased; accordingly, the percentage of S and G2/M subpopulations increased. In contrast, SD-EV had the opposite effect on EC, as depicted in Fig. 4A (left and right panels). This result indicates that HD-EV promoted EC proliferation, while SD-EV retarded the process.

Further, a cell migration assay was performed to assess the effect of EV on EC migration ability. As shown in Fig. 4B, the wound width area significantly decreased in the HD-EV group while increasing in the ascending order of the OFI, MD, and SD-EV groups. This demonstrates that HD-EV enhanced EC motility, while SD-EV impaired the migratory ability.

### Severe dengue plasma-derived EV (SD-EV)-treated CD4^+^ T cells have a marked impact on EC proliferation and apoptosis

The interaction between CD4^+^ T and endothelial cells (ECs) is also essential. Our previous study showed that SD-EV significantly suppresses CD4^+^ T-cell proliferation (20). However, the impact of EV-treated CD4^+^ T cells on ECs has not been studied. Here, we first incubated CD4^+^ T cells with EV isolated from different groups. After 6 days of incubation, the cell supernatant was removed, and CD4^+^ T cells were co-cultured with EC. We observed a significant reduction in the G2-M cell population in EC treated with SD-EV-CD4 group, indicating less proliferation of the EC (Fig. 4C). Also, cell migration assay was performed by giving wound to 70% confluent EAhy926 cells, and images were taken at the 0 h time point. After which, EV-modulated CD4^+^ T cells were added to these wounded EC and incubated for 8 h, and pictures were taken to check the wound width after removal of the supernatant.

Compared with only EV (Fig. 4B), EV-CD4 significantly reduced EC migration. Cells treated with SD-EV-CD4 had increased percent wound width areas (mean% wound width 75% vs 55% in SD-EV) (Fig. 4B and D, right panel).

Compared with the control HD-EV group, the apoptosis rate of ECs was upregulated in OFI-EV, MD-EV, and most significantly with SD-EV (Fig. 4E). At the same time, similar results were also observed in cells treated with SD-EV-CD4. The total early and late apoptosis in the SD-EV-CD4 group was markedly up-regulated and reached 75% (Fig. 4E and F).

The results above demonstrated that HD-EV-treated CD4 cells promoted EC proliferation. In contrast, SD-EV-CD4 cells exhibited the opposite effect and markedly affected EC proliferation and apoptosis.

### SD-EV-treated CD4^+^ T cells induce PD-L1 expression in endothelial cells

Vascular endothelial cells (VECs) cover the innermost wall of arteries, capillaries, and veins and play a decisive role in preserving the barrier function. However, the barrier function can be breached upon interaction with CD4^+^ T cells or prior insult with cytokines, e.g., TNF-α, IFN-γ. The CD4^+^ T cells express a wide variety of co-receptors. Among them, PD-1/PD-L1 interaction with EC has been widely studied (23). Programmed cell death ligand 1 (PD-L1) is a transmembrane protein that binds to PD-1 and thus plays a significant role in the immunosuppressive response.

Enhanced PD-L1 is thought to contribute to the downstream reprogramming of EC activation, affecting vascular integrity (24). In Fig. 1G, we have shown that SD-EV possesses a high level of PD-L1. When interacting with CD4^+^ T cells, inducing PD-1 expression (Fig. 1F). Therefore, we wanted to see if EV only or EV-CD4 can impart signals to ECs through PD-1–PD-L1 interaction.

**Fig 5:**
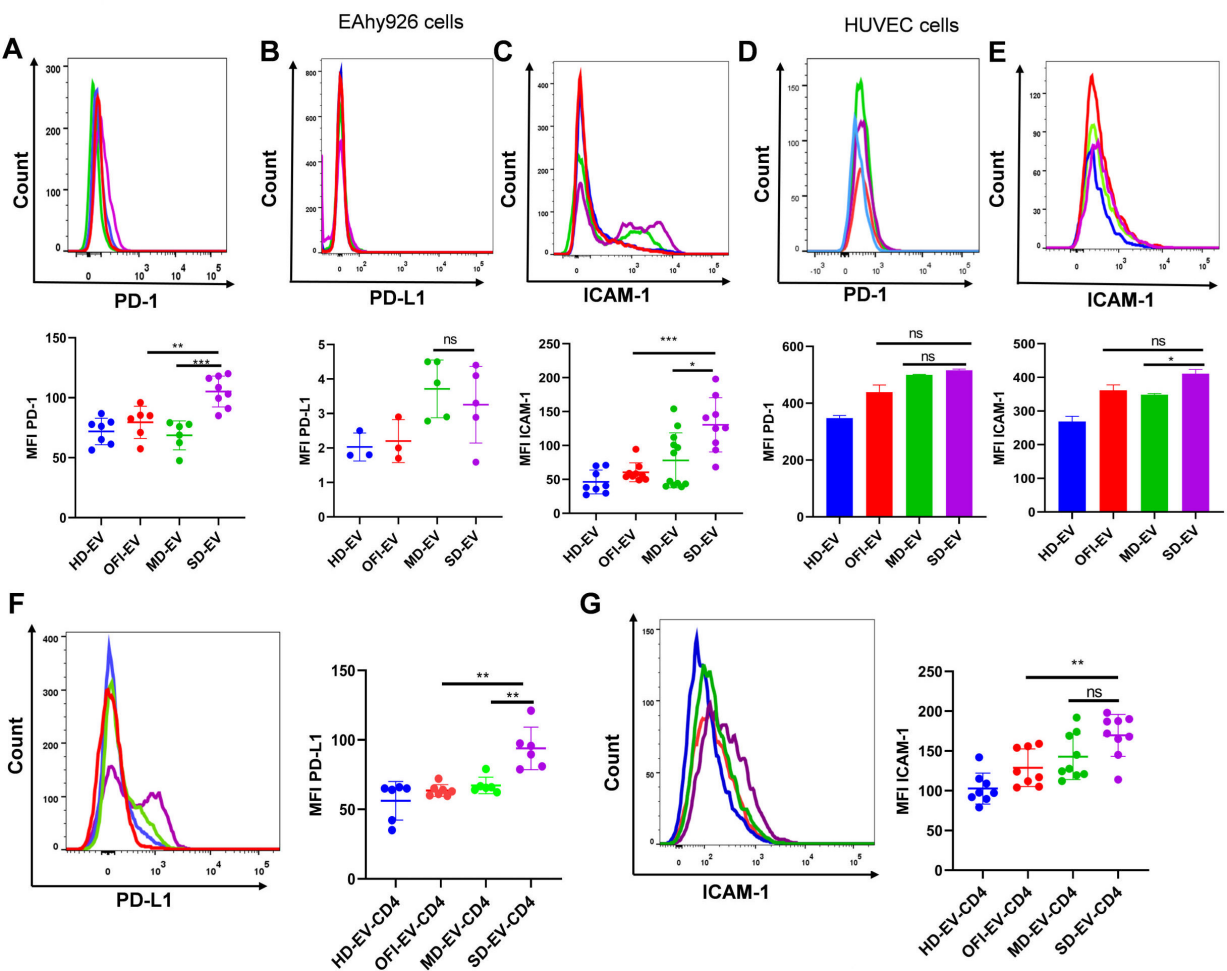
Expression of PD-1–PD-L1 and ICAM-1 on EA.hy926 and HUVEC endothelial cells. (**A–C**) A representative graph of EA.hy926 expression of PD-1, PD-L1, and ICAM after incubating ECs with EVs from all four groups in 20 µg concentration for 18 h. The lower panel shows the combined graph of MFI expression of indicated markers on EC, where each dot represents one set of extracellular vesicles. The Student’s unpaired *t*-test is used to observe the significance. (***P = 0.0002, **P = 0.001, *P = 0.021). (**D and E**) A representative graph of HUVEC expression of PD-1 and ICAM-1 after incubating ECs with EVs from all four groups at 20 µg concentration for 18 h. The bar graphs (lower panel) show the combined graph of increased MFI expression of PD-1 and ICAM-1 by ECs, where each dot represents one set of EV. Student’s unpaired *t*-test is used to calculate the significance. (**F and G**) Representative graphs of EA.hy926 cell expression of PD-L1 and ICAM-1 on EC after incubating EC with EV-modulated CD4^+^ T cells. MFI of indicated markers in the right panel of the graph depicting combined data of MFI. Each dot represents one set of EV-modulated CD4^+^ T cells on endothelial cells (CD4^+^ T cells isolated from three healthy donors and three different sets of EV were used). The Student’s unpaired two-tailed test was applied to get significance (**P = 0.0026).

Human umbilical vein endothelial cells do not exhibit a constitutive expression of PD-L1 (25) under steady-state conditions. However, when we treated the EC with EV isolated from plasma of different study groups, we observed that PD-1 level and cell adhesion molecules ICAM-1 went up significantly on EC (EAhy926) treated with SD-EV (Fig. 5A through C). Intercellular adhesion molecule-1 (ICAM-1) is a cell adhesion molecule that regulates endothelial and epithelial barrier function. ICAM-1 is involved in the adhesion of leukocytes to endothelial cells and is a crucial factor in inflammatory diseases (26, 27). We did not notice a significant increase in PD-L1 expression on EC (EAhy926) treated with EV (Fig. 5B). We observed the same pattern on primary endothelial cells (HUVEC) for ICAM-1 when incubated with EV (Fig. 5D and E). PD-1 levels were upregulated compared with HD-EV on EAhy926 and primary endothelial cells (HUVEC) (Fig. 5A and D); this is probably because SD-EV already had an increased level of IFN-γ and TNF-α, which can increase PD-1 expression on EC (28).

On the contrary, EV interaction with CD4 (EV-CD4) induces PD-1 expression in T cells. Co-culture of EV-CD4 with EC up-regulated PD-L1 expression on EC and increased ICAM-1 expression on EC (Fig. 5F and G).

### Blocking PD-L1–PD-1 interaction reverses EV-CD4-mediated effect on endothelial cells

**Fig 6:**
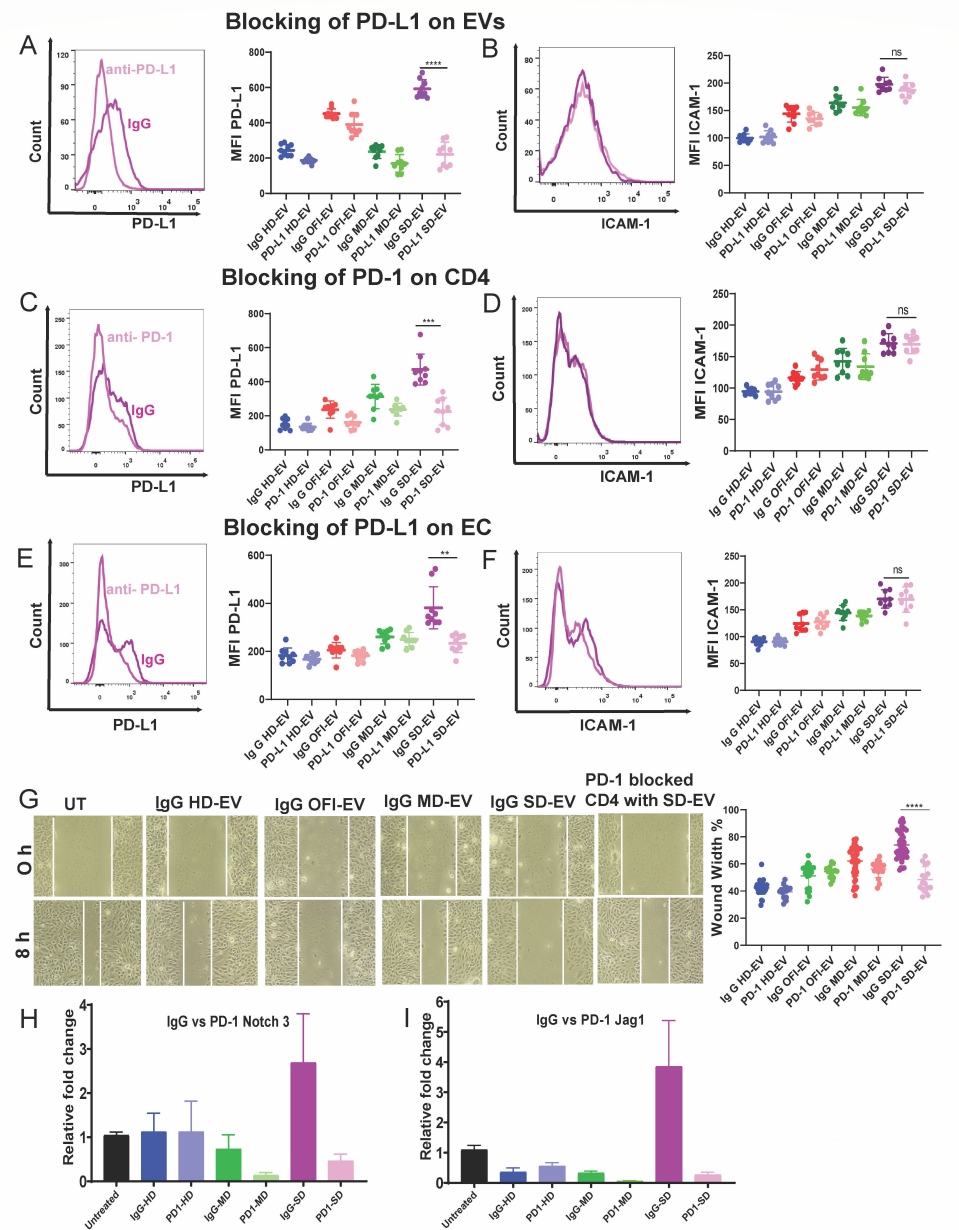
PD-1/PD-L1 axis is critical in EV-modulated CD4^+^ T cell–endothelial cell crosstalk. (**A and B**) Representative histogram of PD-L1 and ICAM-1 expression on endothelial cells (EC) co-cultured with IgG isotype control or PD-L1-blocked EV-modulated CD4^+^ T cells (left panel). Quantified MFI values were plotted for IgG- and PD-L1-blocked groups. The right panel shows a combined graph of MFI of PD-L1 and ICAM-1 with EVs blocked with IgG and PD-L1 with three donors and three different experiments on endothelial cells. Student’s two-tailed unpaired *t*-tests were used (****P < 0.0001). (**C and D**) Representative histogram of PD-L1 and ICAM-1 expression on EC with either EV-modulated IgG isotype control or PD-1-blocked CD4^+^ T cells. Quantified MFI values were plotted in the right panel. Student’s unpaired two-tailed *t*-tests are used. ***P = 0.0003. (**E and F**) Representative histogram of PD-L1 and ICAM-1 expression on EC with Ig G isotype control or PD-L1-blocked endothelial cells co-cultured with EV-modulated CD4^+^ T cells. Quantified MFI values were plotted in the right panel. Student’s unpaired two-tailed *t*-tests are used. (***P = 0.0003; ** *P* = 0.0038). (**G**) Cell migration assay of endothelial cells was performed by creating a wound to these cells by 200 µL tip at 0 h and taking images. These wounded ECs were incubated with EV-modulated IgG isotype control or PD-1-blocked CD4^+^ T cells for 8 h. The quantitative graphs are plotted percentage-wise (right panel). The experiment used EV-modulated CD4 T cells of three different donors. Each dot represents one value of wound width taken at one field of the plate. The Student’s unpaired two-tailed *t*-test is used (****P < 0.0001). (**H and I**) RT-PCR of endothelial cells after incubation with EV-modulated CD4^+^ T cells and EV-modulated PD-1-blocked CD4^+^ T cells for 18 h. Relative fold change of Notch-3 and Jag-1 expression of EC was plotted.

We followed three experimental conditions to understand if PD-1/PD-L1 interaction is needed for PD-L1 and ICAM-1 induction on ECs. First, we blocked PD-L1 on EVs and incubated them with CD4^+^ T cells, and these CD4^+^ T cells were cultured with ECs (Fig. 6A and B). In the second condition, the PD-1 was blocked on CD4^+^ T cells using anti-PD-1 antibody and treated with EV for six days. EV-treated PD-1 blocked CD4^+^ T cells were then incubated with EC (Fig. 6C and D). In the third case, PD-L1 was blocked on EC before incubating with EV-treated CD4^+^ T cells. After 18 h of incubation, PD-L1 and ICAM-1 expression was measured (Fig. 6E and F). As shown in Fig. 6A through F, in all the cases, PD-L1 expression was significantly reduced in the SD-EV treated group; however, no change in ICAM-1 expression was noted, suggesting that ICAM-1 expression in EC, probably following different signaling events. When PD-1 was blocked on CD4^+^ T cells and treated with SD-EV for 6 days, used for cell migration assay on EC, we observed a significant decrease in wound width (Fig. 6G), suggesting that the change in EC migration needed PD-1–PD-L1 interaction.

Notch signaling is the key pathway to controlling different functions of endothelial cells (29). As shown in Fig. 6H, Notch-3 mRNA level increased significantly in EC upon treatment with MD-EV and SD-EV compared to OFI-EV. Blocking of PD-1 on EV-treated CD4 significantly attenuated Notch-3 and Jag-1 transcript expression in EC (Fig. 6I). Together, these data suggest that blocking PD-1 or PDL-1 significantly affects EC properties.

### Blocking of CD44 on SD-EV treated CD4^+^ T cells attenuates ICAM-1 expression on endothelial cells

**Fig 7:**
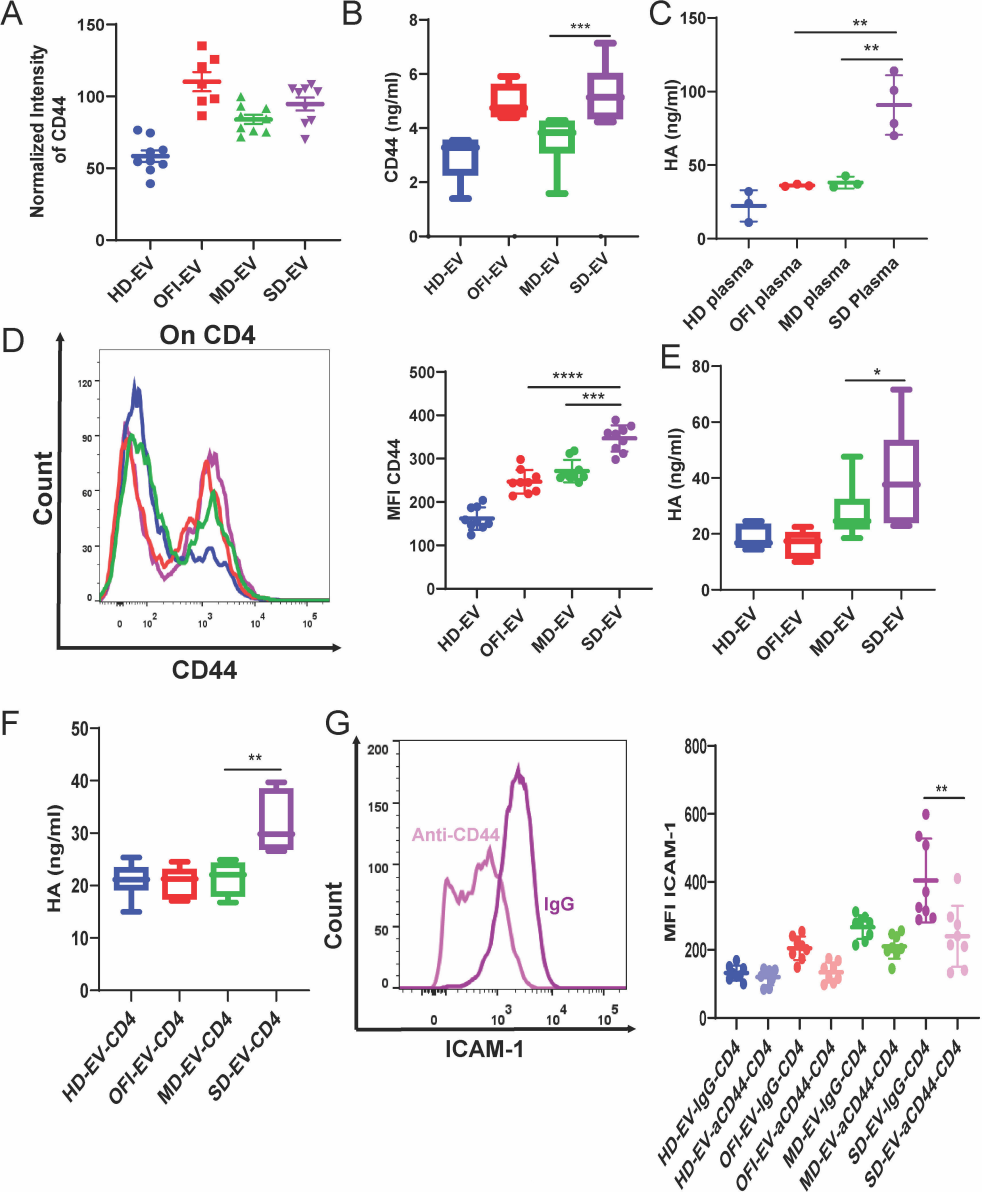
CD44 and ICAM-1 interaction via HA. (**A**) Normalized intensity of CD44 in all four groups of EVs from mass spectrometry data. (**B**) The concentration of CD44 level was measured by ELISA in all groups of EVs and plotted as a box plot. Mann–Whitney test calculates the significance (***P = 0.0008). (**C**) ELISA of hyaluronic acid present in the plasma of healthy donors, other febrile illness patients’ plasma, mild dengue patients’ plasma, and severe dengue patients’ plasma. Mann–Whitney *t*-test is applied (** *P*-value= 0.0021). Each dot represents one set of plasma from different groups. (**D**) Representative image of CD44 expression on CD4^+^ T cells after incubation with EV from all four groups for 6 days. The right panel represents the combined data graph of MFI expression of CD44 on CD4^+^ T cells after incubation with EV. Each dot represents one set of EV. CD4 was isolated from three different donors. Student’s unpaired *t*-test is used to calculate the significance (****P < 0.0001, ***P = 0.0002). (**E**) ELISA of hyaluronic acid (HA) of the endothelial cells incubated with EV alone for 18 h. (*P = 0.0137). (**F**) ELISA of hyaluronic acid of the endothelial cell supernatant after incubating it with EV-modulated CD4^+^ T cells for 18 h. (**P = 0.0012). (**G**) The representative image of ICAM-1 expression on EC after incubating it with EV-modulated CD4^+^ T cells blocked with isotype or anti-CD44 for 18 h. The right panel represents the combined data of MFI expression of ICAM-1 on endothelial cells. Each dot represents one set of EV-modulated CD4^+^ T cells. The Student’s unpaired *t*-test was used to observe the significance (**P = 0.0086). Each dot represents one set of EV-modulated CD^+^ T cells.

Since PD-1/PD-L1 interaction modulation does not affect ICAM-1 expression on EC, we looked for additional receptors on EV and CD4. Apart from PD-L1 expression, our EV proteomics study revealed CD44 enriched in EVs isolated from plasma of OFI, mild, and SD (Fig. 3D and 7A). However, using FACS, we could not detect CD44 expression on the membrane of the EVs (data not shown). However, CD44 can also be available as a soluble cargo form within EV. Indeed, we found that SD-EV and OFI had increased levels of CD44 as determined by ELISA (Fig. 7B). Interestingly, activated CD4^+^ T cells also expressed CD44 expression. CD44 can interact with hyaluronic acid (HA) and release HA in supernatant (30, 31). HA markedly increased ICAM-1 steady-state mRNA and cell surface expression. Also, HA was detected in the plasma of HD, OFI, and mild dengue patients, and HA was found to be present in higher concentrations in severe dengue patients’ plasma (Fig. 7C).

We measured CD44 levels on CD4^+^ T cells upon treatment with EV. We found increased levels of CD44 in CD4^+^ T cells treated with SD-EV than OFI or mild-EV after 6 days post-treatment (Fig. 7D). Since CD44 interacts with HA on endothelial cells, we measured HA level released in endothelial cell culture supernatant upon incubation with EV alone and EV-treated CD4. HA level increased significantly in cell supernatants where EC was treated only with SD-EV or SD-EV-CD4 (Fig. 7E and F). We further checked if CD44 and HA interaction inhibition affected EC’s ICAM-1 expression. We incubated EV-treated CD4^+^ T cells with neutralizing CD44 antibody and put them on ECs. We observed that in CD4 cells treated with HD, OFI, or mild-EV when incubated with EC, there were hardly changes in expression patterns of ICAM-1 on EC (Fig. 7G). However, SD-EV-treated CD4^+^ T cells, when blocked with CD44 antibody, caused a significant reduction in ICAM-1 expression on ECs (Fig. 7G). These data together suggested that increased ICAM-1 expression was probably a consequence of CD44 and HA interaction.

### SD-EV-modulated CD4^+^ T cells secrete a high amount of TNF-α, blocking of which rescues the endothelial cells from apoptosis and high ICAM-1 expression

**Fig 8:**
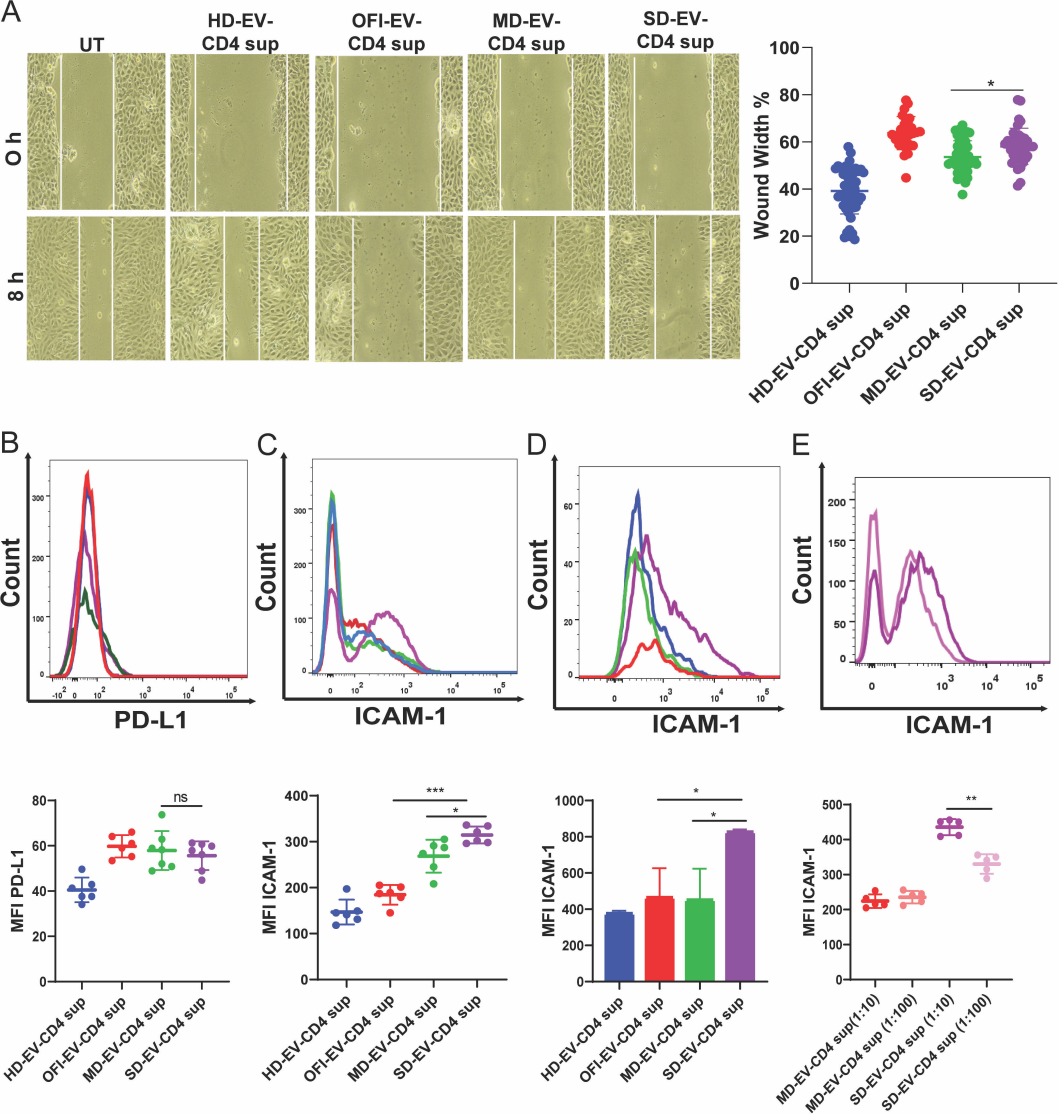
Effect of EV-modulated CD4^+^ supernatant on EC. (**A**) A cell migration assay of endothelial cells (EA.hy926) was performed by giving a wound to these cells by 200 µL tip at 0 h and taking images. These wounded endothelial cells were incubated with EV-modulated CD4^+^ supernatant for 18 h. The combined graph is plotted for the wound width percentage, where the initial wound width was taken as 100%, and after incubation measurements, percentages were plotted. The right panel shows the combined graph of wound width % of different group EV-modulated CD4^+^ T-cell supernatant. The supernatant of three CD4 donors modulated with three different sets of EV was used. Each dot represents one measurement value at one field of the plate. The Student’s unpaired two-tailed *t*-test is used to observe the significance (*P = 0.0048) (**B and C**). A representative histogram showed PD-L1 and ICAM-1 after incubating EC with EV-modulated CD4^+^ T cell supernatant from all four groups for 18 h. The lower panel shows EC’s combined graph of MFI expression of PD-L1 and ICAM-1. The student’s unpaired *t*-test calculates the significance (***P < 0.0001, *P = 0.0184). (**D**) A representative histogram of ICAM-1 incubated with EV-modulated CD4^+^ T-cell supernatant in HUVEC. The bar graph (lower panel) shows the combined MFI expression of ICAM-1 by HUVEC. Student’s *t*-test calculates the significance (*P = 0.0198). (**E**) Representative histogram of ICAM-1 expression on EA.hy926 cells after diluting MD-EV and SD-EV-modulated CD4^+^ supernatant. The lower panel shows the combined graph of decreased MFI of ICAM-1 on diluting the supernatant. Student’s *t*-test is used to observe the significance (**P = 0.0002). Each dot represents supernatant from one individual EV-modulated CD4^+^ T cell.

The key pro-inflammatory cytokines, IFN-α, β, and γ, and TNF-α, are known to be the main drivers for enhanced PD-L1 expression in endothelial cells (32). During inflamed conditions and sufficient IFN-γ, PD-L1 molecules are induced on blood vessel endothelial cells (ECs) to promote crosstalk within filtrating PD-1-expressing T cells (33). We previously reported that when SD-EV was incubated with CD4^+^T cells, several cytokines, including IFN- γ and TNF-α, were released in the supernatant of EV-modulated CD4^+^ T cells (20). Therefore, it will be interesting to see if the supernatant from EV-treated CD4 cells affects ECs. First, we checked the migration ability of ECs in the presence of EV-modulated CD4^+^ T-cell supernatant. As shown in Fig. 8A, compared with HD-EV, supernatant from CD4 treated with OFI-EV, MD-EV, or SD-EV significantly reduced cell migration ability. In another set of experiments, we checked the expression of PD-L1 and ICAM-1 on EC. We observed SD-EV-modulated CD4^+^ T-cell secretome increased PD-L1 expression on ECs similar to OFI and MD-EV-CD4^+^ T-cell supernatant (Fig. 8B). However, ICAM-1 expression was highest on ECs when incubated with SD-EV-treated CD4^+^ T-cell supernatant (Fig. 8C). Similar results were also observed in primary endothelial cells (HUVEC) (Fig. 8D). It could be possible that some soluble factors in the supernatant may induce ICAM-1 expression on EC. We diluted supernatant collected from different groups and treated EC to test this hypothesis. As shown in Fig. 8E, the ICAM-1 level decreased significantly in the SD-EV-treated CD4^+^ Tcell supernatant group with increasing dilution.

**Fig 9:**
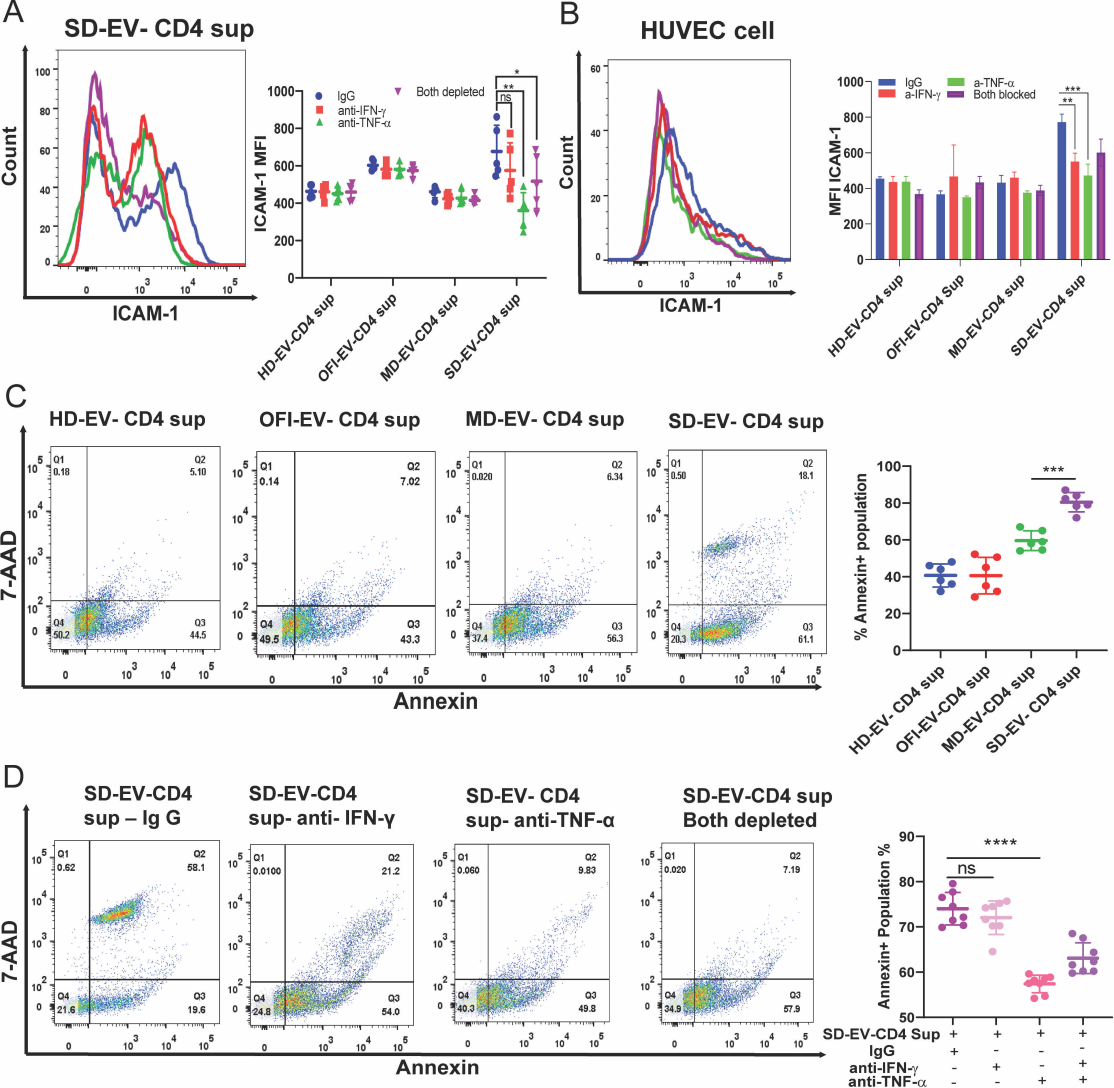
TNF-α in SD-EV-modulated CD4^+^ T-cell supernatant critical for enhanced ICAM-1 expression and apoptosis in endothelial cells. SD-EV-modulated CD4^+^ T-cell supernatant was incubated with anti-IFN-γ (0.03 µg/mL), TNF-α (0.04 µg/mL), or both to deplete the respective cytokines. The cytokine-depleted supernatant was added on EA.hy926 and HUVEC cells. Representative image of ICAM-1 expression on EA.hy926 (**A**) and HUVEC (**B**) after incubating with SD-EV-modulated CD4^+^ T-cell supernatant depleted for the cytokines. The right panel shows the MFI for ICAM-1 in different groups. ANOVA was used to calculate the significance. **P = 0.008. (**C**) Representative FACS images of EA.hy926 cells for apoptosis after incubating with indicated EV-modulated CD4^+^ T-cell supernatant. The right panel shows the combined graph of % Annexin positive EA.hy926 cells. Each dot represents one set of EV-modulated CD4^+^ T-cell supernatant. Student’s unpaired two-tailed *t*-test was used to calculate the significance (***P < 0.0001). (**D**) EA.hy926 cells were incubated with cytokine-depleted supernatant (IFN-γ, TNF-α, or both) obtained from indicated EV-modulated CD4^+^ T cells. Representative FACS images of EA.hy926 cells for apoptosis. The right panel shows the combined graph of % Annexin positive EA.hy926 cells. Each dot represents one set of EV-modulated CD4^+^ T-cell supernatant. Student’s unpaired two-tailed *t*-tests were used to observe the significance (**** *P* < 0.0001, ns = 0.2977).

**Fig 10:**
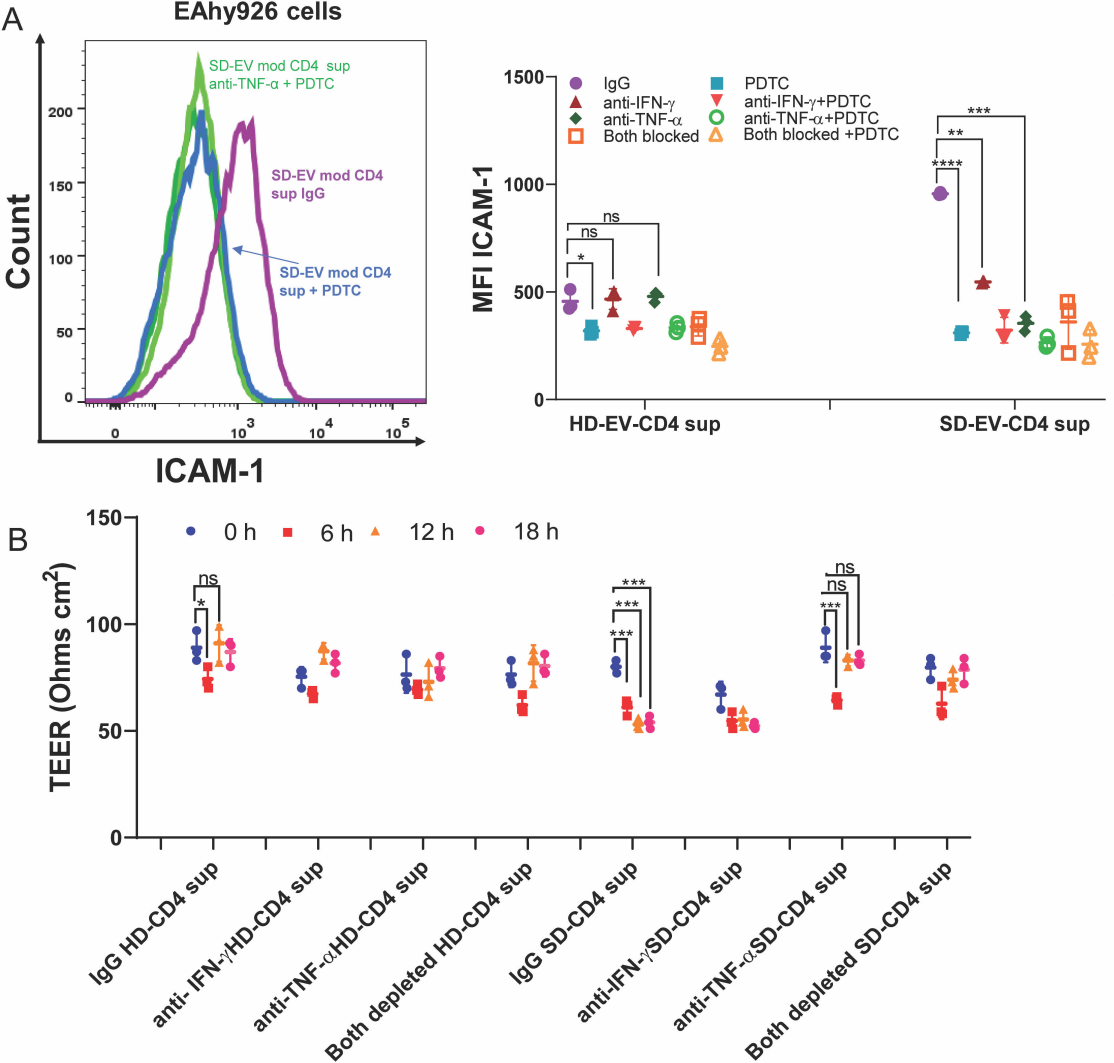
Induction of ICAM-1 by TNF-α is NF-κB mediated. (**A**) Representative histogram of ICAM-1 MFI expression of EA.hy926 in the presence of PDTC (NF-κB inhibitor) with EV-modulated CD4^+^ T-cell supernatant and cytokine-depleted (IFN-γ, TNF-α, and both) supernatant. The right panel shows the combined MFI of ICAM-1 of EC after incubating with healthy (HD) and severe (SD) EV-modulated CD4^+^ T-cell supernatant and cytokine-depleted supernatant. Student’s unpaired *t*-test is used. Each dot represents one set of EV-modulated CD4^+^ T-cell supernatant (IgG, IFN-γ, or TNF-α blocked). (**B**) Transendothelial electric resistance (TEER) of EA.hy926 cell monolayer is shown at different time points, 0, 6, 12, and 18 h, after incubating with healthy (HD) and severe (SD) EV-modulated CD4^+^ T-cell supernatant and cytokine-depleted supernatant. Each dot represents one set of EV-modulated CD4^+^ T-cell supernatant (IgG, IFN-γ, or TNF-α blocked). The Student’s unpaired *t*-test is used (***P = 0.0001).

As we have shown earlier, the SD-EV supernatant contained significantly increased levels of IFN- γ and TNF-α; we used neutralizing antibodies against IFN- γ and TNF-α to deplete the cytokines from the supernatant. The specific cytokine-depleted supernatant from SD-EV-CD4, when added to EC, ICAM-1 expression was attenuated significantly in TNF-α-depleted supernatant. However, such reduction was not observed in IFN- γ-depleted supernatant (Fig. 9A), suggesting that TNF-α is probably driving ICAM-1 expression on EC. Similar observations were found on primary endothelial cells; TNF-α-blocked cytokine showed higher reduction than IFN-γ-blocked cytokine (Fig. 9B). Further, SD-EV-treated CD4^+^ T-cell supernatant significantly augmented apoptosis compared with MD-EV or OFI-EV-treated CD4 supernatant (Fig. 9C). Interestingly, apoptosis was significantly reduced when we used TNF-α-depleted supernatant from the SD-EV-CD4 group. However, no significant decrease in apoptosis was noticed in cells treated with IFN-γ depleted supernatant from the SD-EV-CD4 group (Fig. 9D, right panel). Thus, our data suggested that an increased level of TNF-α released from SD-EV-treated CD4^+^ T cells is critical for increased ICAM-1 expression and apoptosis in ECs. TNF-α induced ICAM-1 expression may be due to activation of NF-kB pathway (34). To explore this possibility, we treated the cells with NF-κB inhibitor (PDTC) for 2 h,, and supernatant or TNF-α- or IFN-γ -blocked supernatant was added on ECs. After 18 h of incubation, ICAM-1 was measured. Pre-treatment with NF-κB inhibitor significantly reduced ICAM-1 expression upon treatment with SD-EV-modulated CD4 supernatant (Fig. 10A). NF-kB inhibitor treatment did not have an additional effect on TNF-α- or IFN-γ-blocked supernatant-mediated effect on ECs (Fig. 10A). In a separate experiment, transendothelial electrical measurement (TEER) was measured upon incubation with supernatant or blocked cytokines. As compared with HD-EV, SD-EV incubated CD4-derived supernatant significantly reduced TEER measurement within 6 h of incubation and constantly reduced it at later time points. Whereas HD-EV-CD4-released supernatants started increasing TEER value at later time points (Fig. 10B). Our data suggest that SD-EV-modulated CD4^+^ T cells released TNF-α, caused endothelial leakiness, and increased ICAM-1 expression via the NF-kB pathway.

## DISCUSSION

**Fig 11:**
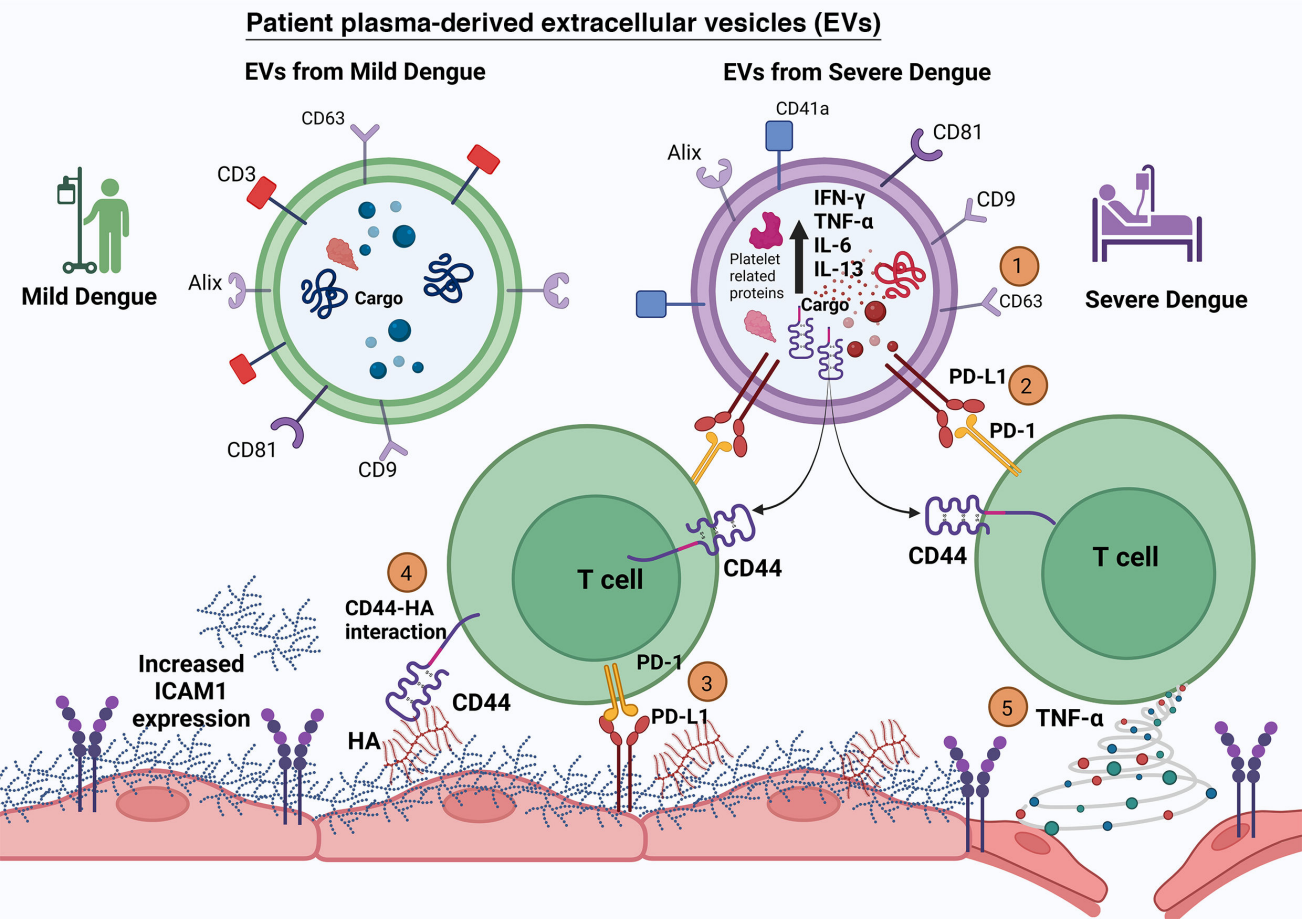
Graphical representation of the study, representing EV-CD4 interaction and EV-CD4-EC interaction (1). CD63 (abundant marker) was found to be present on the surface of EVs (2). PD-L1 on the SD-EV surface induces the expression of PD-1 on CD4^+^ T cells, inhibits its proliferation, and produces cytokines (20) (3). PD-1 on CD4^+^ T cells induces PD-L1 on endothelial cells; this pathway leads to significant changes in endothelial cell properties like cell migration ability (4). SD-EV induced the expression of CD44 on CD4^+^ T cells; CD44 interacts with hyaluronic acid (HA) on the endothelial cells, which results in the release of HA in the supernatant and unmasking of ICAM-1 on endothelial cells (5). The presence of TNF-α in the supernatant from SD-EV-CD4^+^ T cells resulted in high apoptosis and high ICAM-1 expression of endothelial cells. Taken together, all these, EV, EV-modulated CD4^+^ T cells, and their supernatant-induced changes in endothelial cell properties.

Extracellular vesicles (EVs) are vital in viral infection and disease pathogenesis (35). Most studies reported EV isolated from dengue-infected different cell types (36). The EV either carries viral NS1 (37) or disrupts the monolayer integrity of endothelial cells (38). In our study, we isolated EVs from the plasma of dengue-infected patients. We compared their effect on ECs against EVs isolated from the plasma of OFI or mild and severe dengue patients. Our previous studies reported the modulation of CD4^+^ T cells by EV isolated from the plasma of severe dengue patients. We found that PD-1 was expressed at significantly higher levels on Th1 cytokine-producing CD4^+^ T cells when exposed to SD-EV (20). We further extended our study and looked into the effect of EV-CD4^+^ T cells on endothelial barrier dysfunction. Using EA.hy926 cells and HUVEC cells, we have demonstrated that (i) severe dengue plasma-derived EV (SD-EV)-treated CD4^+^ T cells have retarded proliferation and impaired the migratory ability of endothelial cell (EC); (ii) SD-EV-CD4^+^ T cell induces PD-L1 expression on endothelial cells. Blocking of PD-1 on CD4 significantly hindered PD-L1 expression on EC and increased EC migration ability. (iii) SD-EV induces CD44 expression on CD4^+^ T cells, and on co-culture with EC, ICAM-1 expression is induced, and hyaluronic acid is detected in the supernatant of EC, indicating endothelial barrier dysfunction. Blocking of CD44 significantly reduced ICAM-1 expression on endothelial cells. (iv) SD-EV-treated CD4 T cells secreted a high level of TNF-α in the supernatant, which results in the induction of ICAM-1 expression on EC, reducing TEER and enhancing apoptosis via the NF-kB pathway (Fig. 11).

CD4^+^ T cells exhibit increased and sustained PD-1 expression during bacterial infection in mice and humans (39). Even in dengue-infected patients, increased expression of PD-1 was reported (16). Human umbilical vein endothelial cells (HUVEC) do not exhibit a constitutive expression of PD-L1 under steady-state conditions. However, PD-L1 gets upregulated when treated with IFN-γ and TNF-α (25), indicating that key pro-inflammatory cytokines, IFN-α, β, and γ, and TNF-α are the main drivers for enhanced PD-L1 expression on endothelial cells. In our study, we observed increased PD-L1 expression on endothelial cells upon co-culture with PD-1-positive-activated CD4^+^ T cells. The exact mechanisms for CD4-mediated EC activation are not precise. However, blocking of PD-1 inhibits PD-L1 and Notch expression on ECs, suggesting that PD-1-positive CD4^+^ T cells induce Notch signaling to enhance PD-L1 expression on ECs. Studies indicate that Notch signaling is considered to be the homeostatic regulator of the endothelium by enabling ECs to (i) resist apoptosis, (ii) arrest the cell cycle and promote EC’s life span, and (iii) build up the functional blood vessels from a damaged vessel (40, 41). Notch pathway activation upregulates the expression of PD-1/PD-L1 and may play a novel role(s) in maintaining pulmonary EC barrier regulation. PD-L1^high^ cells equally tend to overexpress the Notch-3 molecules, where down-modulation of Notch-3 proteins hampered PD-L1 expression in pulmonary vascular ECs (24), indicating a strong link between optimal levels of notch signaling and PD-L1 expression. Simultaneously, it could be speculated that activated CD4 can secrete TNF-α and IFN-γ, which in turn can induce PD-L1 expression on ECs (Fig. 8B). Interestingly, we observed that PD-L1 modulation did not correlate with increased ICAM-1 expression. We demonstrated that SD-EV-CD4 expressed PD-1 and CD44 on its surface. SD-EV-CD4, when interacting with ECs, induces PD-L1 and ICAM-1 expression on ECs. However, if we blocked PD-1 on CD4, it attenuated PD-L1 expression on EC but did not inhibit ICAM-1 expression on EC. However, when we blocked CD44 on SD-EV-CD4 and cultured it with EC, it inhibited the ICAM-1 expression on ECs. It seems that CD44 interacted through HA on EC and induced a signal that increased ICAM-1 expression. CD44 and HA interaction is evident in CD4^+^ T cells and EC interaction (42–44). We showed that SD-EV-CD4-derived secretome contains TNF-α and depletion of TNF-α from secretome significantly reduced ICAM-1 expression compared with HD-EV-CD4. Interestingly, blocking of NF-kB pathways with PDTC inhibitor significantly reduced ICAM-1 expression both in HD-EV-CD4 secretome-treated EC and SD-EV-CD4 secretome-treated EC, suggesting that ICAM-1 expression induced through secretome via NF-kB pathway. Anti-TNF-α therapy is reported to cause improvement in endothelial dysfunction in rheumatoid arthritis, COVID-19, and sepsis (45–47). Anti-TNF-α therapy significantly reduces mortality even in the experimental dengue infection model (48). Consistent with this observation, TNF-α depletion from secretome reduced EC apoptosis. However, another study warns against anti-TNF-α therapy in COVID-19 patients as it could augment the SARS-CoV-2 receptor and increase the risk of infection through Notch-1 signaling (49). Therefore, more studies are required before concluding anti-TNF-α therapy for dengue-infected patients.

We tried to detect viral RNA or protein (NS1) in EV of dengue-infected patients’ plasma. However, we could not detect viral RNA and protein in the EV (Fig. 1E). In our study, as compared with mild dengue, where 12% of patients were reactive for dengue IgG, about 40% of severe dengue patients were reactive for dengue IgG, confirming secondary infection is more in severe dengue. It could be possible that circulatory EV composition may be different in severe dengue due to the increased percentage of secondary infections in the severe group that may also impact PD-1 expression on CD4^+^ T cells. A study published by Haltaufderhyde et al. reported that the number of activated (PD-1+) CD8 and CD4 T cells were highest during the critical phase of illness and significantly higher in patients with secondary versus primary dengue infections (16).

Overall, our study highlighted the significant importance of circulating EV. Depending on the disease condition, EV significantly impacted CD4 and EC behaviour. We also demonstrated that SD-EV alone or SD-EV-treated CD4 or their secretome can cause EC damage. In conclusion, we provided evidence that SD-EV modulated CD4 carrying PD-1 and CD44 when interacting with EC significantly affected endothelial properties through direct interaction or via secretome and may be significant in dengue-mediated endothelial dysfunction.

## MATERIALS AND METHODS

### Clinical samples

Blood samples were collected from the patients and tested for the DENV NS1/IgM and IgG, as described earlier (50). The clinicians categorized dengue patients as per WHO criteria. A total of 90 dengue patients (male = 61, female = 29, ages between 18 and 58  years), including severe dengue patients (SD; *n* = 65; NS1/IgM+/IgG+ = 27, only NS1/IgM+ = 38, duration of fever (days) at the time of recruitment = 3–7 days; platelet counts = <30,000 per mm^3^ of blood), mild dengue patient (MD; *n* = 25; NS1/IgM+/IgG+ = 3, only NS1/IgM+ = 22; duration of fever (days) at the time of recruitment = 2–5 days; platelet counts = >100,000 per mm^3^ of blood), were included in this study. Also, plasma from dengue negative other febrile illness (OFI; *n* = 45, male = 32, female = 13, age between 20 and 55 years Dengue NS1/IgM-, RT-PCR negative; duration of fever (days) at the time of recruitment = 1–4 days; platelet counts = >50,000 per mm^3^ of blood) and healthy donors’ (HD; *n* = 65 age between 24 and 30 years) were included in this study.

### Isolation of EV from the ultracentrifugation process

Plasma from mild (MD), severe dengue (SD), other febrile illnesses (OFI), and healthy donors (HDs) were collected individually. For EV isolation, five samples (200 µL each) were pooled to make 1 mL plasma and diluted with filtered PBS to make a total volume of 11 mL. EV isolation was performed using a multistep ultracentrifugation process. Briefly, cell debris was removed by centrifugation at 2,000 rpm for 30 min and differentially ultracentrifuged to obtain extracellular vesicles free from cellular debris as previously described (20).

### Nanoparticle tracking analysis (NTA)

The EV were diluted (1:1,000) in filtered 1× PBS and injected into a laser chamber for NTA by NanoSight LM20 (Malvern Instruments Company, NanoSight, Malvern, UK). The following settings were used for data acquisition: camera level, 11; acquisition time, 60 s; and detection threshold, 3. Each particle’s Brownian motion was tracked thrice between frames, and the size was calculated using the Strokes–Einstein equation.

### Transmission electron microscopy

Approximately 4–5 μL of EVs was placed on Formvar/carbon-coated pre-glow discharged copper grids (#CF300-CU/50) and allowed to adsorb for 2 min in a dry environment. The grid was then washed in RNase-free water thrice for 20, 40, and 60 s, stained with 1.5% Phospho-Tungstic acid for 50 s, and placed in a clean environment for drying. The grid was observed at the 80 k threshold under the transmission electron microscope (JEOL- JEM 1400 flash, JEOL, USA).

### Western blot of EV

The protein concentration of the isolated extracellular vesicles was determined using the micro-BCA protein assay kit (#23235, Thermo Pierce, Rockford, IL, USA) following the manufacturer’s instructions. Briefly, EVs were lysed in reducing sample buffer containing DTT, SDS, and beta-mercaptoethanol (BME) separated on 10% SDS/PAGE gels (Bio-Rad), always applying 20 µg protein/lane and transferred onto an Immobilon-P PVDF membrane (EMD Millipore) for Western blotting. Membranes were blocked with 5% BSA, followed by overnight incubation at 4°C with different antibodies of tetraspanins present in EVs. The following antibodies were used: CD63 AB 216130 (Abcam, Cambridge, UK), calreticulin (Cat # PA1-903, Invitrogen), and dengue NS1 (Cat# PA5-32207, Invitrogen). HRP-conjugated secondary antibody incubated for 1 h at room temperature, and after giving three washes of PBST, blots were developed by using an ECL detection kit (# 32132 Thermo, Waltham, USA)

### Extracellular vesicle isolation and surface marker characterization

To detect the surface marker on EVs derived from OFI, MD, and SD groups, flow cytometry was done by using 4 µm aldehyde/latex beads (#A37304, Thermo, Waltham, MA USA). Then, 20 µg of protein equivalent EVs was incubated in 5 µL of beads overnight, and excess beads were removed. As described earlier, 10% BSA was added to block unspecific sites for 30 min (20). The beads were stained with antibodies specific to CD63 (CD63-BV421 # 740080, BD Biosciences San Diego, USA) and PD-L1 (PE-C7, **#** 25–5983-42, Thermo Pierce, Rockford, IL, USA) and data were acquired using FACS Verse on the low threshold and analyzed by FlowJo v10 (FlowJo LLC).

### RT-PCR of vero and C6/36 cell line for viral RNA

Vero cell and C6/36 cell lines were used to detect the presence of viral RNA in EV isolated from severe dengue patients’ plasma samples. Vero and C6/36 cells were seeded in MEM and L-15 media, respectively. Upon 70% confluency, EVs were added in 20 µg concentration, and the virus alone (MOI of 0.2) and virus with SD-EV were also taken as positive control. Dengue virus serotype 2 (DV-2) virus was prepared using the C6/36 cell described in (51). After 4 days of incubation, cells were harvested, and RNA was extracted using TRIzol reagent (Invitrogen), according to the manufacturer’s instructions. The dengue viral genome was amplified using previously described primers (51).

### Sample preparation for mass spectrometry

The isolated extracellular vesicles were quantified and dissolved in 100 mM ammonium bicarbonate (ABC). Then, 100 µg of the dissolved EV was aliquoted from each sample, followed by a reduction in 10 mM DTT at 56°C for 30 min and alkylation with 20 mM IAA at RT for 1 h. Further, the reduced and alkylated proteins were digested with a 1:15 ratio of trypsin (Pierce, Thermofisher) at 37°C for 18 h. Digests were then desalted using a C18 silica cartridge (Waters) and dried in speed-vac. The dried pellet was resuspended in Solvent A (2% Acetonitrile,0.1% formic acid) before injecting in LC-MS.

### Mass spectrometric analysis of peptide mixture

Tryptic digests from each sample were analyzed using a ZenoTOF 7600+ (AB Sciex) linked with a Waters microscale LC system (ACQUITY UPLC M-Class System) for data capture in SWATH mode. An equivalent quantity of digested peptides (1 µg) was placed into the Luna 5 µm C18 Micro trap column (20 × 0.3 mm, 100 Å, Eksigent). SWATH data were recorded in triplicate. The tryptic peptides were resolved using a nanoEase M/Z HSS T3 analytical column (150 × 300 µm, 1.8 µm, 100 Å, Waters) at a flow rate of 5 µL/min in a linear gradient of solvent B (100% (v/v) acetonitrile with 0.1% (v/v) formic acid) for 22 min, for a total run duration of 32 min. A SWATH acquisition strategy was used with 65 overlapping variable-size windows covering a mass range of 400–1250 Da and an accumulation period of 20 ms with a cycle time of 1.74 s.

### Data processing and data availability

The acquired .wiff files from SWATH-MS were analyzed with Spectronaut pulsar 18 (Biognosys). A *de novo* spectral library was generated from the .wiff files of the acquired samples using UniProtKB human protein database for protein identification. The generated spectral library was then used to search the SWATH-MS files. The FDR for precursor and peptides was set to 1%, and MS2 level interference correction was enabled. Global and dynamic parameters were employed for the normalization strategy and XIC RT extraction, respectively. The enzyme specificity was set for trypsin with a maximum missed cleavage of 2. Carbamidomethyl on cysteine was used as a fixed modification, whereas oxidation of methionine and N-terminal acetylation were considered variable modifications. For subsequent analysis, the extracted ion peak intensities of peptides corresponding to the identified protein groups were log2 transformed, normalized, and processed via statistical *t*-test to identify unique, differentially regulated proteins in the exosome samples. Further, for downstream analysis, the raw extracted matrix from Spectronaut containing PGquant values for the identified proteins was processed in R software (R 4.3.1) and metaboanalyst 5.0 web tool for data representation.

### Endothelial cell and CD4^+^ T cell co-culture experiments

Blood was withdrawn from healthy donors in EDTA vials, with prior consent and diluted with an equal volume of DPBS (cat no. #2381367, Thermo Waltham, MA USA). PBMCs were isolated by using lymphoprep (#07851, Stem cell, Vancouver, Canada), layering on a Sepmate tube (Cat no. #15415 Stem cell, Vancouver, Canada), and centrifuged at 800×*g* for 20 min with break off. Based on negative selection, CD4^+^ T cells were isolated from the purified PBMCs using a CD4^+^ T cell isolation kit (# 17952 Stem cell, Vancouver, Canada). CD4^+^ T cells (2 × 10^5^ cells/well) were seeded in 96-well U-bottom plates. Cells were activated with CD3:CD28 activator (Immunocult Stem Cell #10991 Vancouver, Canada). Then, 20 µg protein equivalent concentration of EV from all four groups in multiple sets was added and incubated in a CO_2_ incubator for 6 days. After 6 days, EV-modulated CD4^+^ cells were centrifuged, counted, and seeded with endothelial cells in a 1:1 ratio for different experiments. Simultaneously, cell supernatant was collected, and 20 µg equivalent supernatant was used to treat endothelial cells for other experiments.

### Cell migration assay

EA.hy926 cells (ATCC-CRL-2922) were cultured in a DMEM incubator with 5% CO2 at 37°C. EA.hy926 cells were seeded in 24-well plates in DMEM media with exo-depleted FBS. After attaining 70% confluency, a scratch was made using a 200 µL pipette tip. Images of scratch were taken at 0 h using a light microscope. EVs (20 µg protein equivalent concentration) were added from all four groups and incubated for 8 h for migration studies. After 8 h, images were retaken to observe cell migration. Values of wound closure were measured, and the percentage of wound closure was calculated.

In another set of experiments, the EV-modulated CD4^+^ T cells (EV-CD4) and cell supernatant, as described in the previous section, were added separately in the scratched endothelial cell plates. After 8 h of incubation, wound healing was assessed by using microscopy.

### Cell cycle assay

EA.hy926 (2 × 10^5^ cells/well) cells were seeded in 24-well plates in DMEM media, and upon confluency, EV alone (20 µg concentration) and EV-modulated CD4^+^ T cells (EV-CD4) (1:1) were added and incubated for 18 h. After incubation, the supernatant was removed, trypsin was added, and cells were harvested for cell cycle analysis by propidium iodide (PI) staining (#P4170, sigma). Then, 70% chilled ethanol was added dropwise and incubated for 30 min on ice to fix the cells; after 30 min, cells were washed twice by adding PBS and centrifugation at 12,000 rpm for 15 min. After washing, the resultant pellet was re-suspended in 200 µL of fresh PI (50 µg/mL) and incubated for 20 min. FACS was done to analyze different phases of the cell cycle.

### Expression of PD-1/PD-L1 and ICAM-1 on endothelial cells

Endothelial cells (EA.hy926 and HUVEC) were seeded at 0.4 × 10^6^ in 24-well plates and cultured with exo-depleted media; after attaining 70% confluency, EVs were added and incubated for 18 h to assess the MFI of PD-1, PD-L1, and ICAM-1 marker expression on them. Cells were harvested by adding trypsin and centrifuged at 12,000 rpm for 15 min, and PD-L1 (PE-C7) (**#** 25–5983-42, Thermo Pierce, Rockford, IL, USA)**,** ICAM-1 (PE) (# 12-0549-42, Thermo Pierce, Rockford, IL, USA), and PD-1 (BV-421) (#AB205921, BD Bioscience’s San Diego, USA) were added. Similar experiments were done with endothelial cells after incubating cells with EV-modulated CD4 (EV-CD4) cells or with EV-CD4 cell-derived cell supernatant for 18 h. After the incubation, the cell supernatant was removed. Endothelial cells were trypsinized and centrifuged at 12,000 rpm for 15 min, and analyzed for PD-1, PD-L1, and ICAM-1 expression by FACS.

### Apoptosis assay

Endothelial cells (EA.hy926) were seeded at 0.4 × 10^6^ cells in 24-well plates in DMEM (#AL007A). After attaining 70% confluency, EV or EV-CD4 or EV-CD4 supernatant was added and incubated for 18 h to assess their apoptotic status by adding FITC Annexin and 7-AAD (cat no- 640922, BioLegend). Cells were harvested using trypsin and centrifuged at 12,000 rpm for 15 min, then antibodies were added. Ten thousand events were counted on flow cytometry. Analysis performed by Flowjo software.

### IFN-γ and TNF-α blocking in EV-CD4^+^ T-cell supernatant

The supernatants collected from EV-CD4^+^ T cells were incubated either with anti-IFN-γ (#AF-285-SP) (0.03 µg/mL) and anti-TNF-α (#MAB610-SP) (0.04 µg/mL) (R&D Systems) neutralizing antibodies or with isotype control antibodies for overnight at 4°C. After incubation, IgG/IgA beads were added for 2 h at RT, and supernatants were centrifuged for 15 min at 4°C at 12,000 rpm to remove bead-bound IFN-γ and TNF-α. Specific cytokine-depleted supernatants were used to treat endothelial cells for 18 h, and PD-L1/ICAM-1 expression, apoptosis assay, and TEER measurement were performed.

### PD-L1 blocking on EV

To block PD-L1 on the EV surface, purified EV samples (20  µg protein equivalent) were incubated with PD-L1 blocking antibodies (10  µg/mL) or IgG isotype antibodies (10  µg/mL) in 200 µL PBS and then washed with 10 mL PBS and pelleted by ultracentrifugation to remove the un-bound free antibodies. Then, these blocked EVs were used on CD4^+^ T cells. After 6 days of incubation, CD4^+^ T cells were harvested, counted, and seeded with ECs in a 1:1 ratio. PD-L1 and ICAM-1 expressions were measured by FACS after 18 h of incubation. A scratch was made for the wound healing experiment before adding CD4^+^ T cells using a 200 µL tip. After 8 h of incubation, wound healing was assessed by using microscopy.

### PD-1 and CD44 blocking on CD4^+^ T cells

Purified CD4^+^ T cells were incubated either with PD-1 blocking antibody (10 µg/mL) (#149989-80, Thermo Pierce, Rockford, IL, USA) or CD44 blocking antibody (30 µg/mL) (#MA4400, Thermo Pierce, Rockford, IL, USA) or IgG isotype antibody (10 µg/mL) in 200 µL of PBS for 2 h at RT. Washing was done twice in DPBS to remove excess antibodies. Cells were re-suspended in 10% exo-depleted FBS containing RPMI, and 0.2 × 0^6^ cells were seeded on a 96 U-bottom plate. Activator (CD3:CD28) and EV from different groups were added to the respective wells. Cells were harvested after 6 days and co-cultured with EA.hy926 in a 1:1 ratio, and PD-L1, ICAM-1 expression, and cell migration assay were performed as described above. In another experiment, RNA was isolated from EC after 18 h incubation, and RT-PCR for Notch-3 and JAG-1 was performed using primers described below. Notch-3- (Forward) 5′- TTTGAGGGTCAGAATTGTGAAGTG-3′,(Reverse) 5′-TCGGTGTCCTGGACAGTCG-3′. Jag-1 (Forward) 5′-GAAGCAGAACACGGGGCGTT-3′ (Reverse) 5′-CAGGTCACGCGGATCTGAT-3′.

### PD-L1 blocking on endothelial cells

PD-L1 blocking was done on endothelial cells before adding EV-CD4^+^ T cells. PD-L1 blocking antibodies (10  µg/mL) ( #149989-80, Thermo Pierce, Rockford, IL, USA) or IgG isotype antibodies (10  µg/mL) in 200  µL PBS was added on EC and incubated for 2 h. Unbound free antibodies were removed by multiple washing with PBS. EV-CD4^+^ T cells were added in a 1:1 ratio. PD-L1, ICAM-1 expression, and cell migration assay were performed as described above.

### CD44 and hyaluronic acid (HA) measurement using ELISA

The plasma of healthy donors, OFI, mild, and severe dengue patient plasma was used to detect CD44 (CD44 antigen ELISA test kit, #EH0654, FineTest) and HA (#EU2556 HA ELISA kit, FineTest). The supernatant of endothelial cells cultured with EV from all four groups and EV-modulated CD4^+^ T cells were also included in the study. According to manufacturer guidelines, 20 µg of equivalent protein concentration samples was diluted with assay buffer and added to the ELISA plate. The observation was taken at 560 nm. Values were calculated by subtracting the value of the blank and quantified against the standard.

### Transendothelial electrical resistance (TEER) measurement

Approximately 2 × 10^5^ EA.hy926 cells/well were seeded in 24-well plates in DMEM media, and upon confluency, TEER measurements were taken at the 0 h time point through a Millicell ERS Voltohommeter (MERCK). EV-CD4^+^ T-cell supernatant was added in 20 µg concentration. At different time points, TEER was observed by cleaning the probe with 70% ethanol and dipping both probes into the media of the 12-well chamber plate.

### NF-κB inhibition

EA.hy926 (2 × 10^5^ cells/well) were seeded in 24-well plates in DMEM media. Upon 70% confluency, NF-κB inhibitor-pyrrolidine dithiocarbamate (PDTC) (25 µM) (# P8765, Sigma) was added. After 2 h, cells were centrifuged for removal of excess PDTC, 20 µg equivalent concentration of the EV-modulated CD4^+^ T-cell supernatant, either IgG treated or specific cytokine-blocked supernatant (IFN-γ and TNF-α) was added on endothelial cells. After 18 h, supernatant was discarded, trypsin was added, and cells were harvested for ICAM-1 expression through FACS.

### Statistical analysis

Each experiment is performed on three individual healthy donors with three sets of different EV to obtain the pattern and reduce variability. Every dot represents the data of one donor by the one set of EV samples. ANOVA with multiple comparisons and unpaired student’s *t*-tests were mainly used for the comparative perspective. Statistical analysis was performed using GraphPad Prism, and FACS analysis was performed using FlowJo X software. A *P*-value of < 0.05 was considered statistically significant.

## Data Availability

The proteomics data are available via ProteomeXchange with the identifier PXD058037.
